# Supplementary figures and images for: Human brain state dynamics are highly reproducible and associated with neural and behavioral features
Source: PLoS Biol. 2024 Sep 24;22(9):e3002808. doi: 10.1371/journal.pbio.3002808 (PMC11421804; doi:10.1371/journal.pbio.3002808)

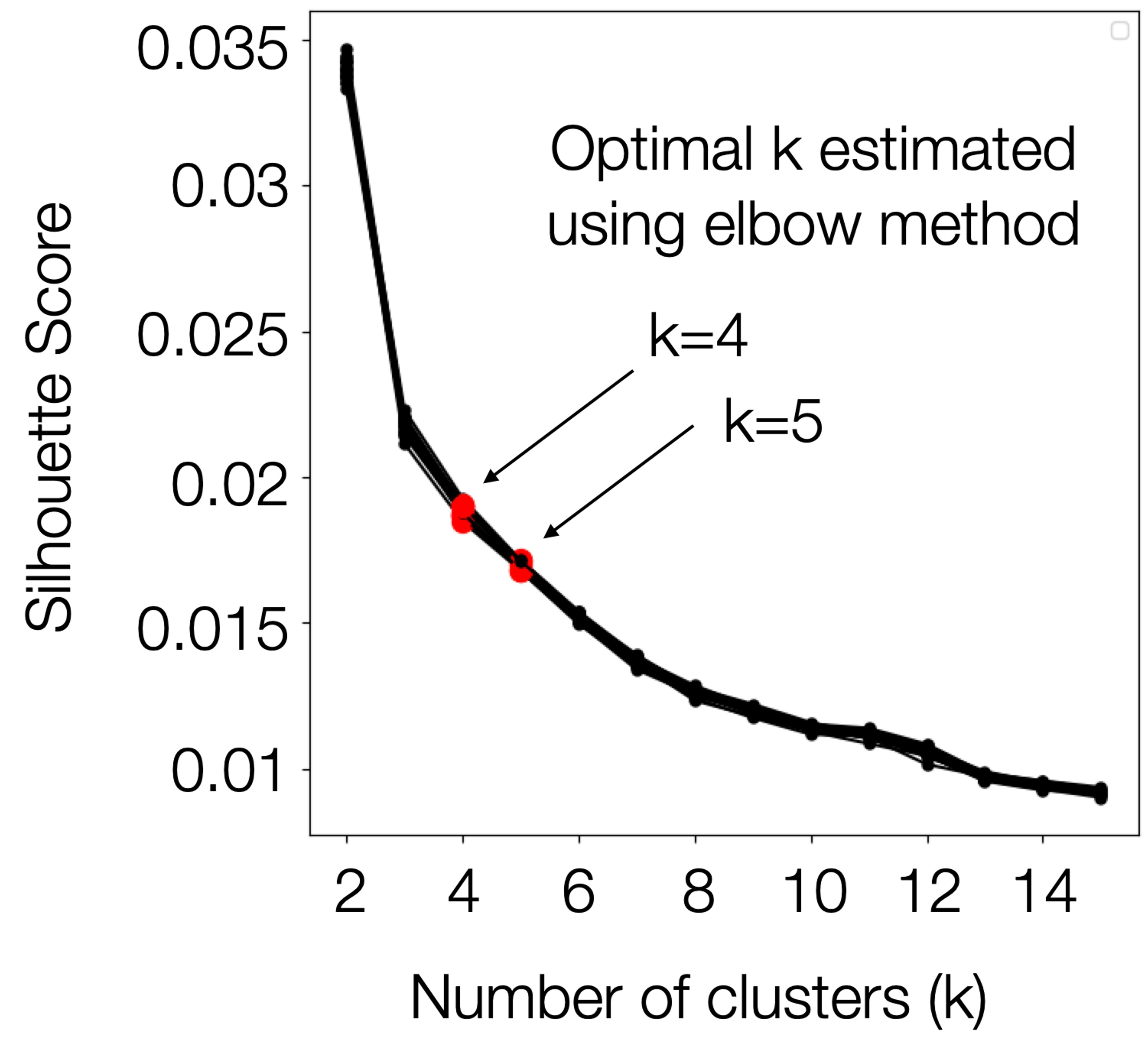

Supplement: S2 Fig — Silhouette scores were estimated across different numbers of clusters (k) from the K-means clustering solution from a split data. Results from 10 permutations (2 split-halves in each permutation) are shown. Optimal k values were estimated using the elbow method for the Silhouette scores and are highlighted in red. (TIF) [file pbio.3002808.s002.tif]

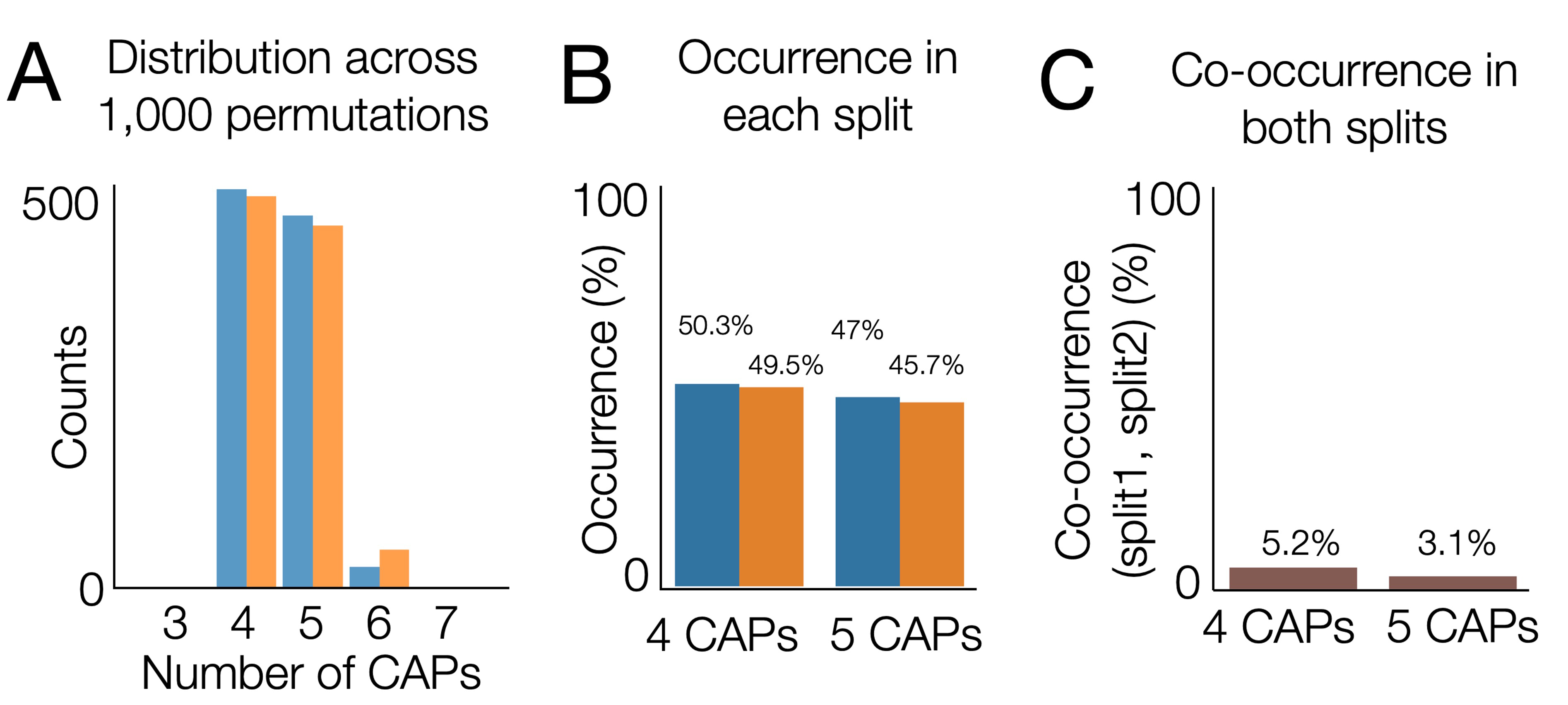

Supplement: S3 Fig — (A) The estimated number of CAPs (k) in each split across 1,000 permutations. (B) Occurrence rate (%) of k = 4 or k = 5 solutions in each split. (C) Co-occurrence rate (%) of k = 4 or k = 5 solutions in both splits. (TIF) [file pbio.3002808.s003.tif]

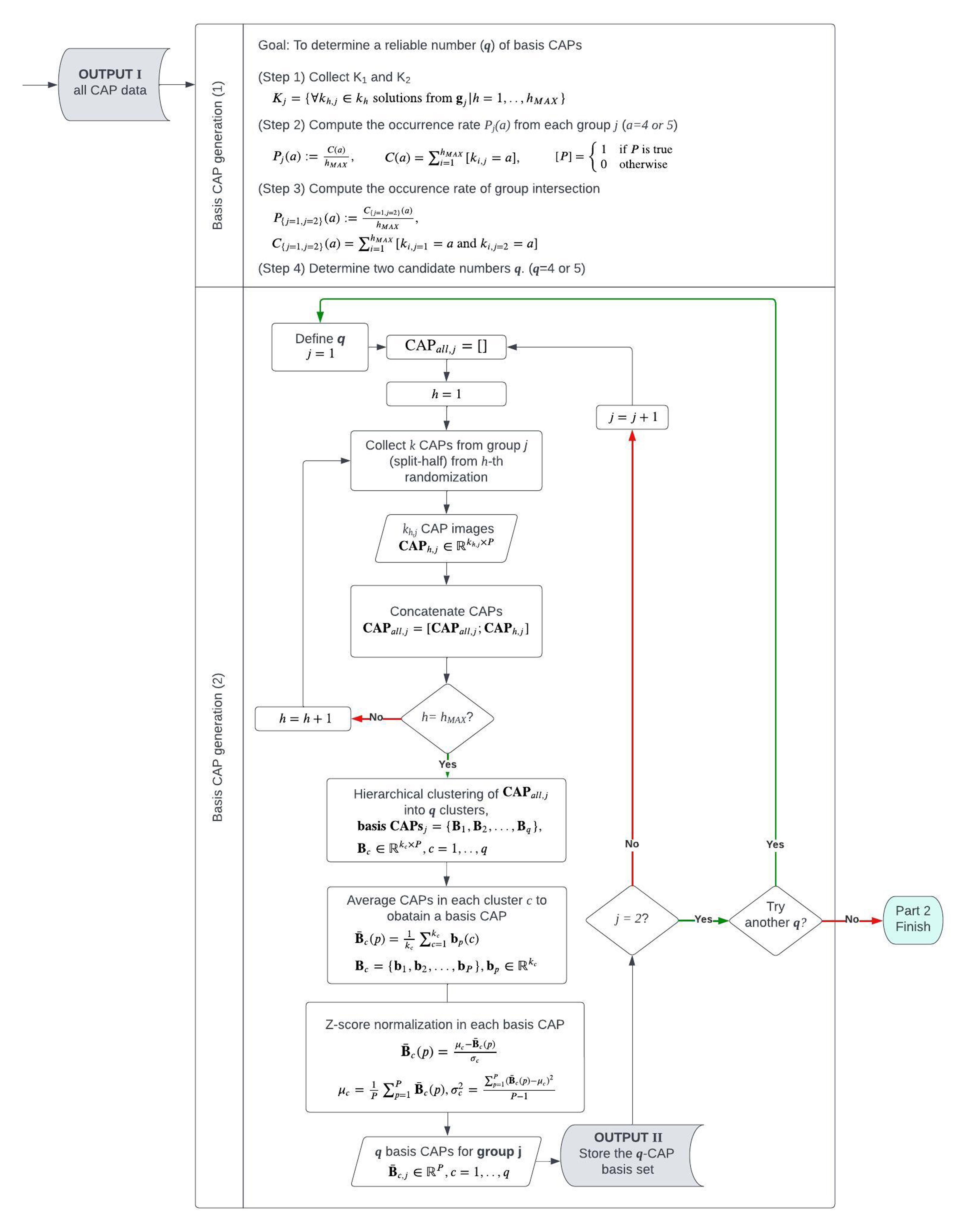

Supplement: S4 Fig — (TIF) [file pbio.3002808.s004.tif]

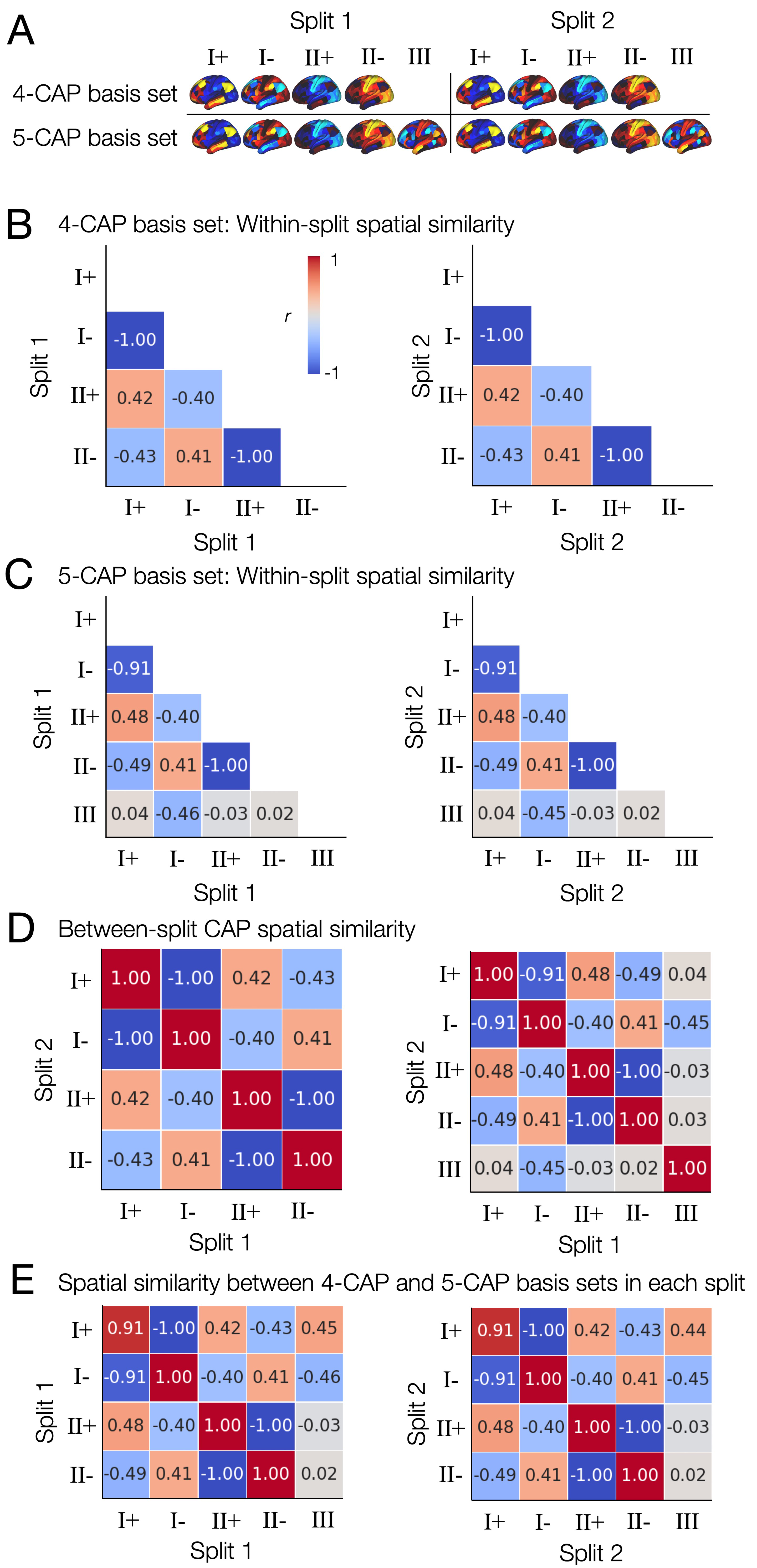

Supplement: S5 Fig — (A) Spatial patterns of the basis CAPs in each split-half data. The 4-CAP basis set and the 5-CAP basis set were generated independently from the same split-half data, using the hierarchical clustering across 1,000 shuffled split-half resampling, as described in S2 Fig. (B) Spatial similarity (r, correlation coefficient) of the 4-CAP basis set within the split 1 data (left) and within the split 2 data (right). r values were rounded to the nearest 2 decimal digits for visualization. (C) Spatial similarity of the 5-CAP basis set within the split 1 data (left) and within the split 2 data (right). (D) Spatial similarity of the 4-CAP basis set between the split 1 and 2 data (left) and of the 5-CAP basis set between the split 1 and 2 data (right). (E) Spatial similarity between the 4-CAP basis set and the 5-CAP basis set within the split 1 data (left) and within the split 2 data (right). (TIF) [file pbio.3002808.s005.tif]

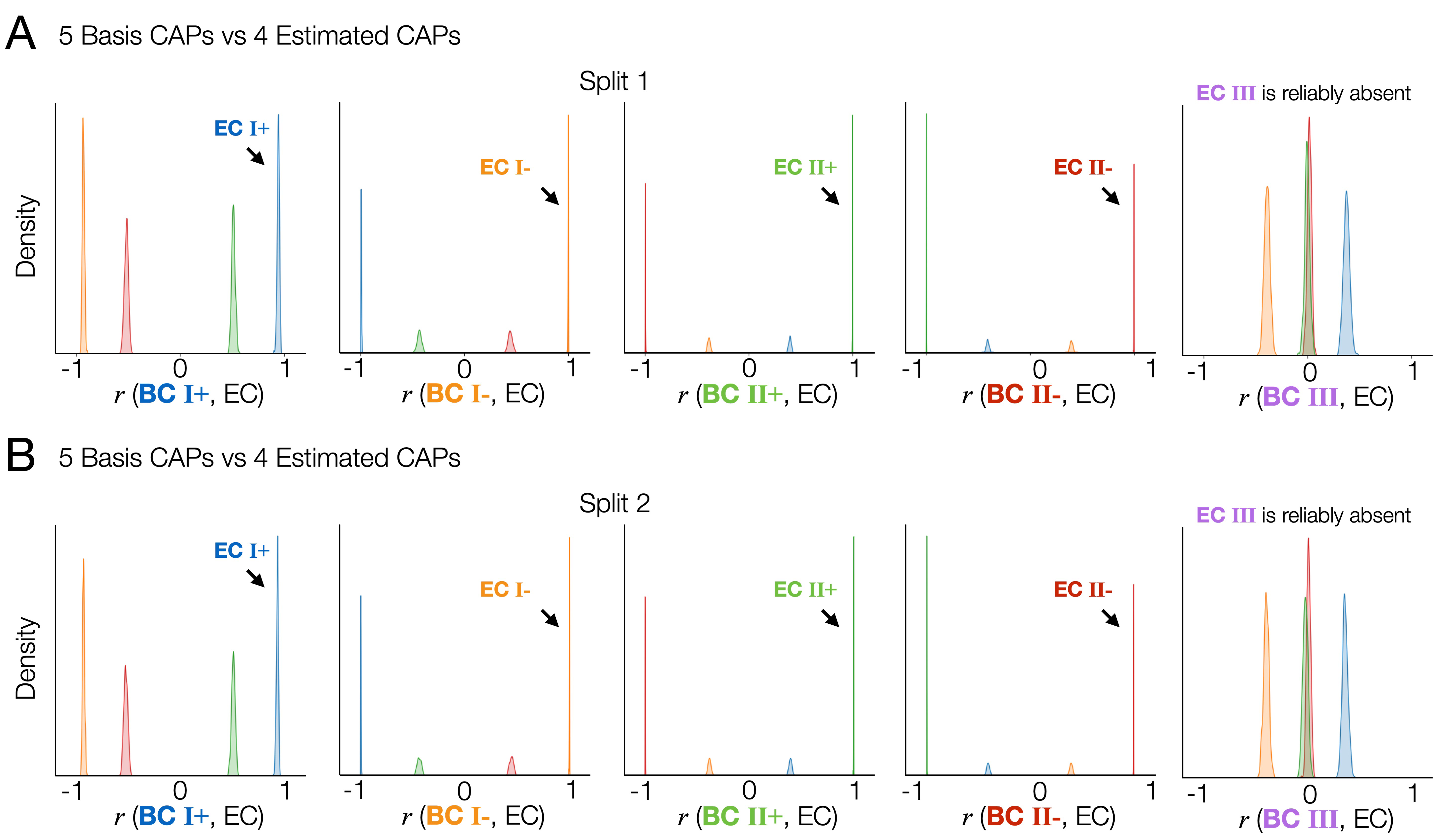

Supplement: S7 Fig — (TIF) [file pbio.3002808.s007.tif]

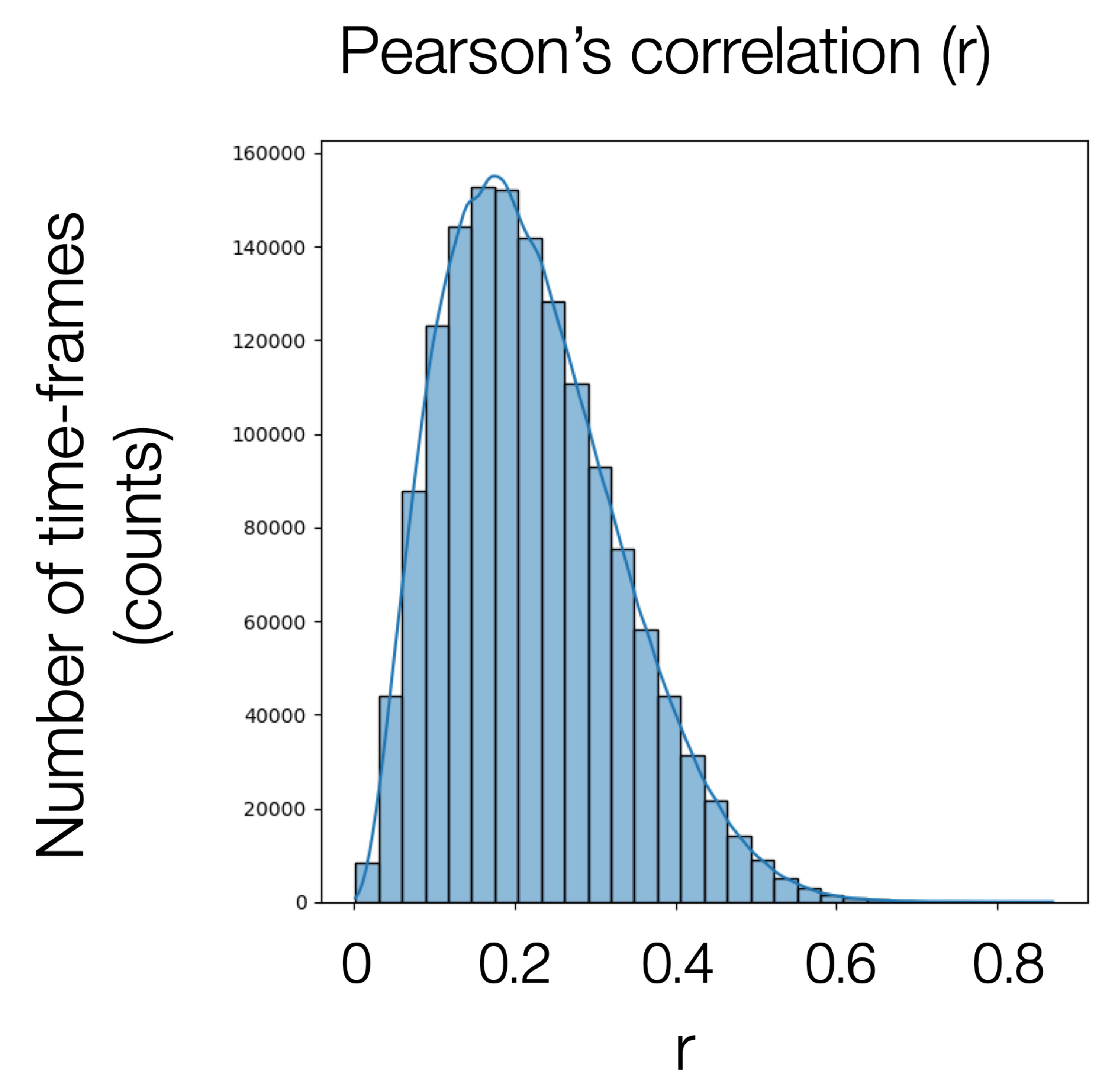

Supplement: S8 Fig — (TIF) [file pbio.3002808.s008.tif]

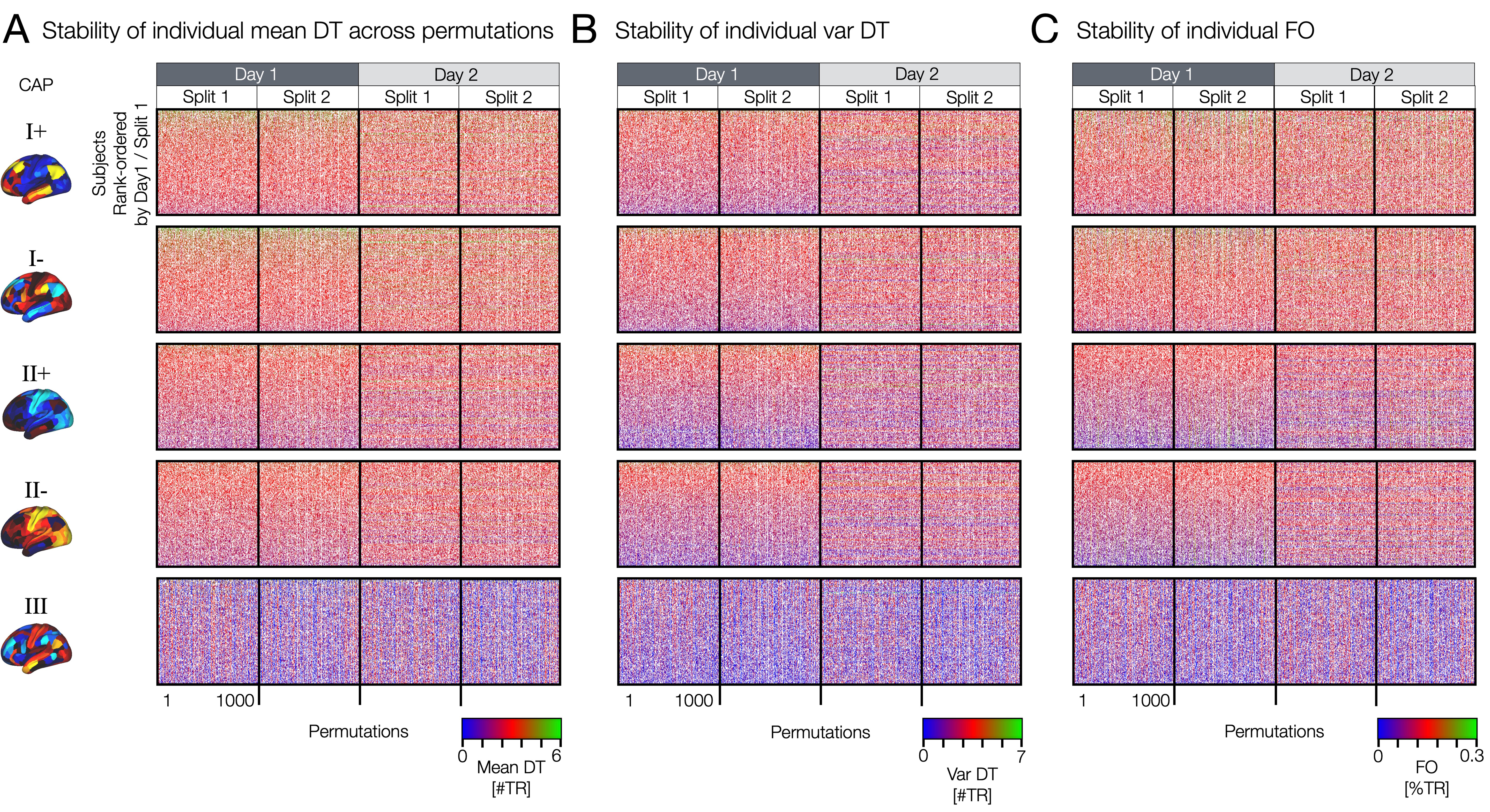

Supplement: S9 Fig — (TIF) [file pbio.3002808.s009.tif]

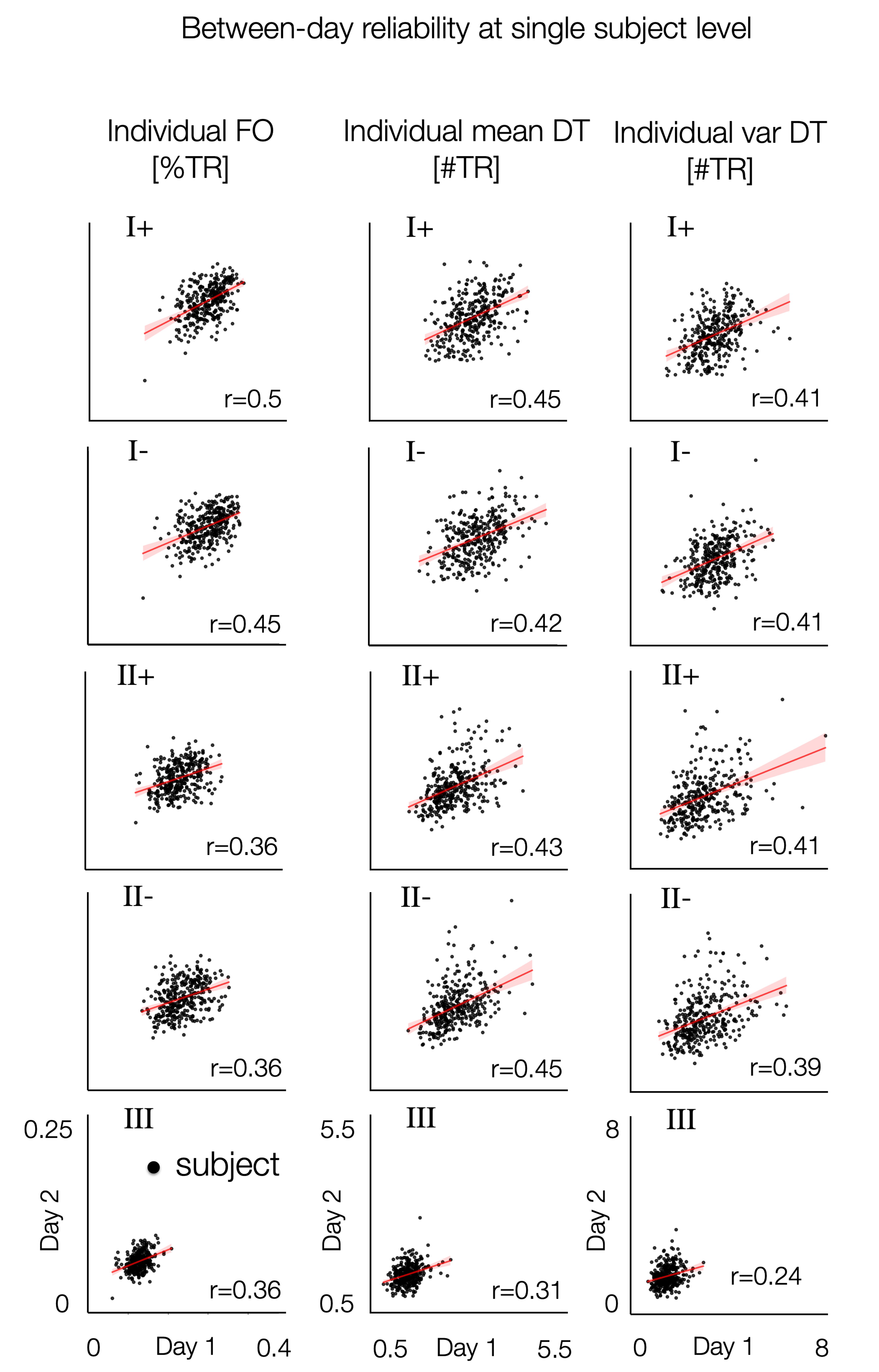

Supplement: S10 Fig — Each datapoint in the scatter plot is a subject. For each subject, neural measures were averaged across permutations. (TIF) [file pbio.3002808.s010.tif]

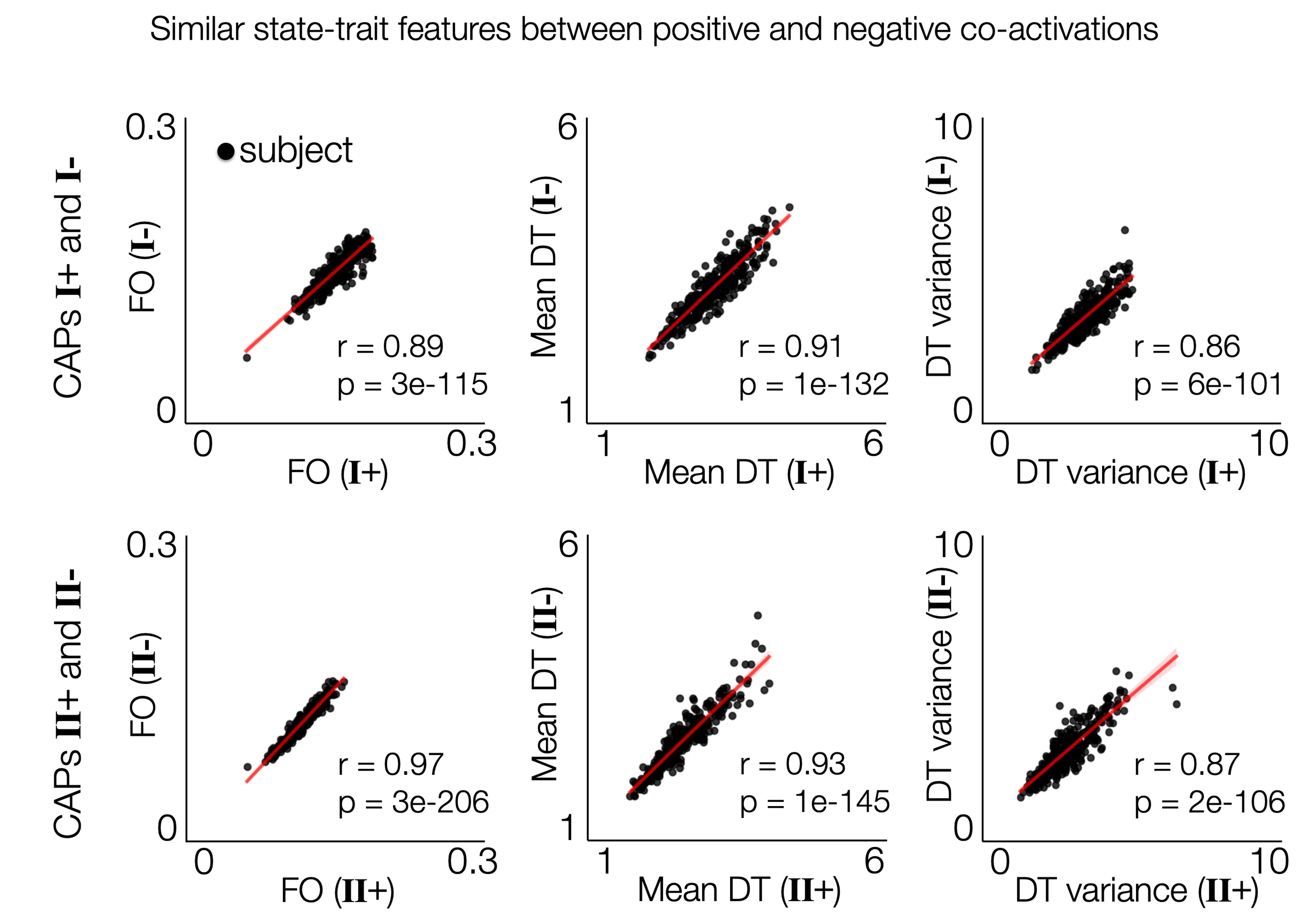

Supplement: S11 Fig — CAP states I and II have similar FO, mean DT and DT variance across the positive and negative co-activation states (I+ vs. I- and II+ vs. II-). Each data point indicate a subject. The temporal metric values across all permutations and 2 days were averaged within each subject. (TIF) [file pbio.3002808.s011.tif]

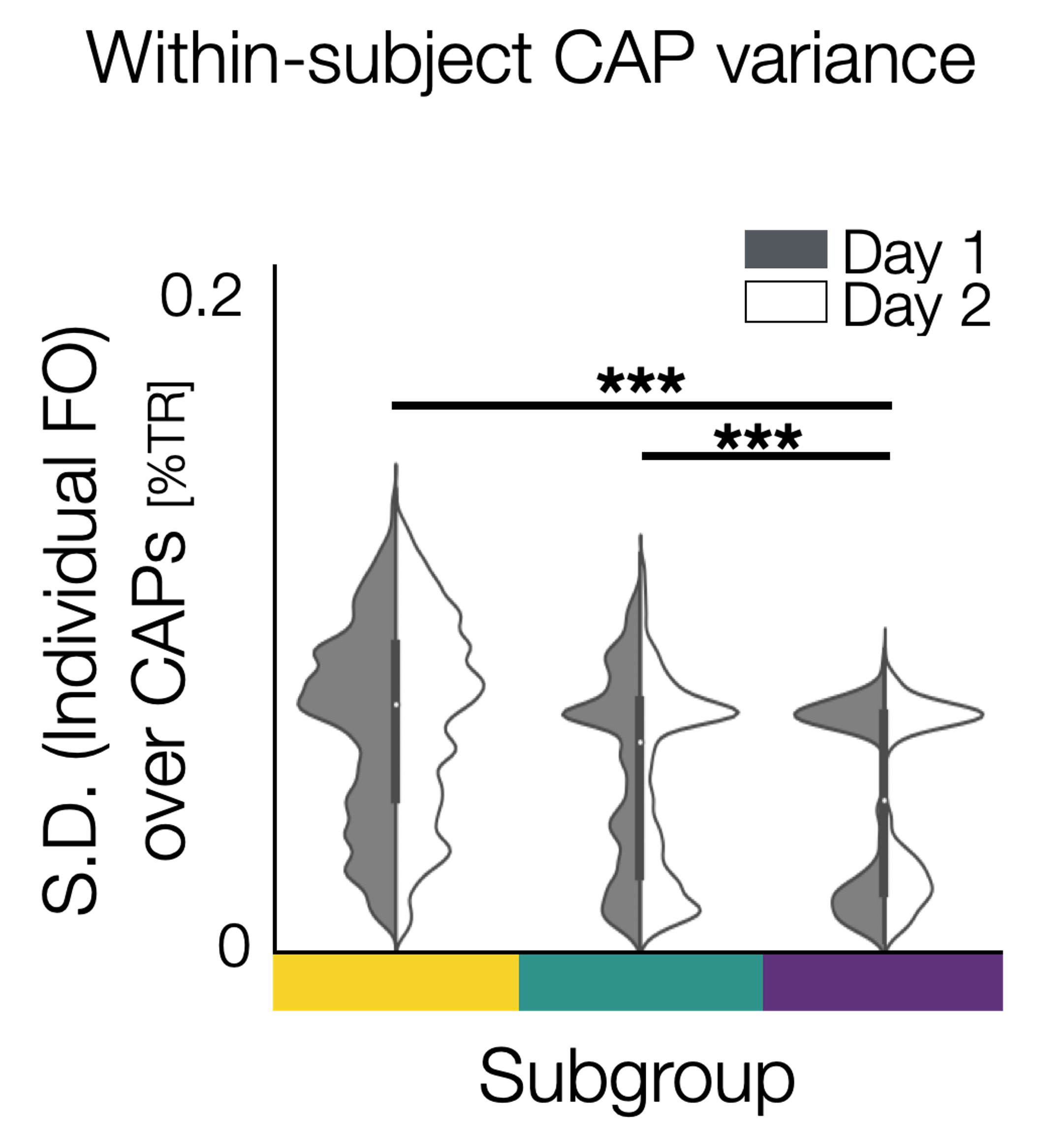

Supplement: S12 Fig — (TIF) [file pbio.3002808.s012.tif]

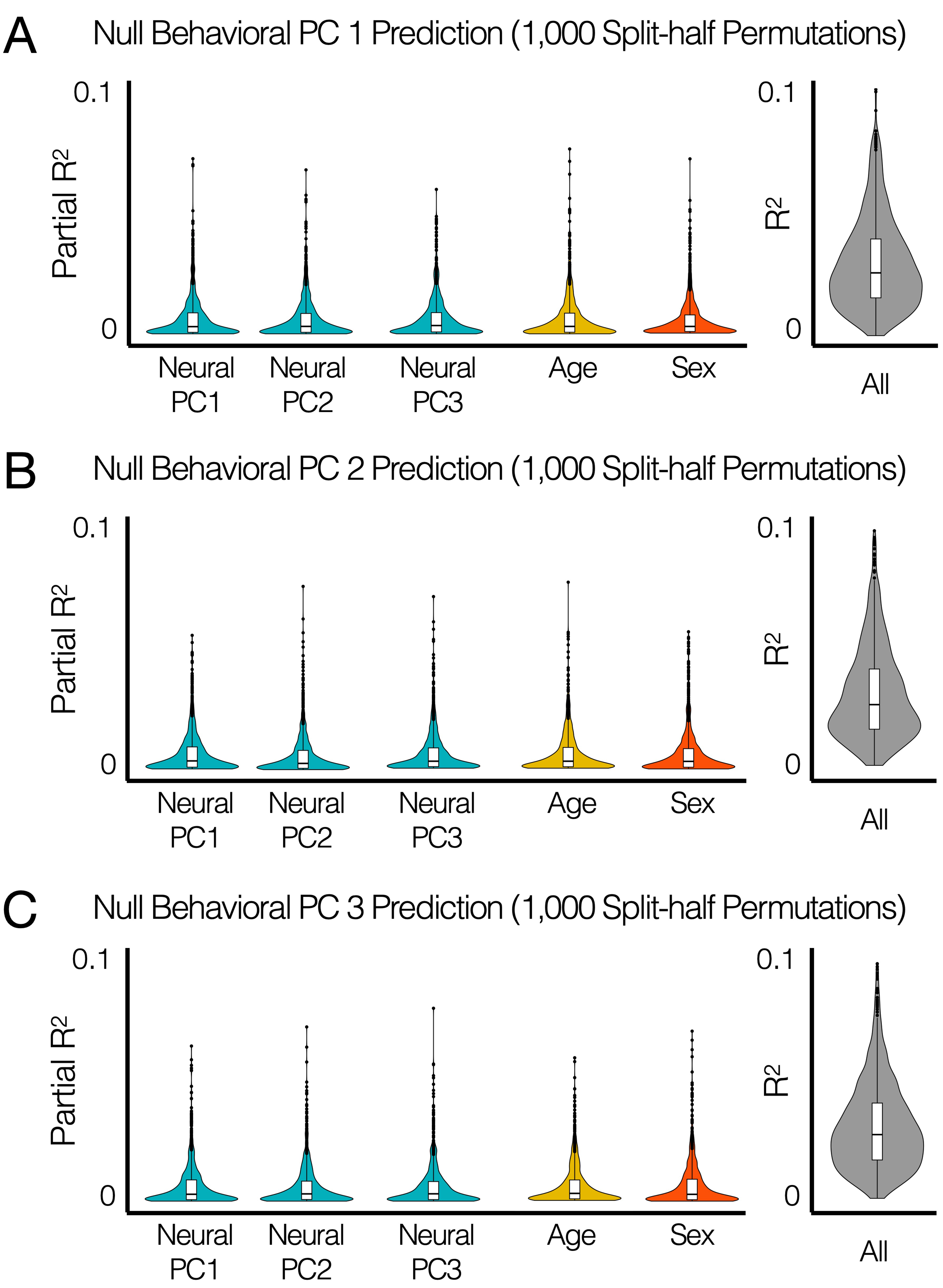

Supplement: S15 Fig — Null distributions of partial R2 were estimated for each predictor in the neuro-behavioral association model trained from a split data across 1,000 split-half permutations. (TIF) [file pbio.3002808.s015.tif]

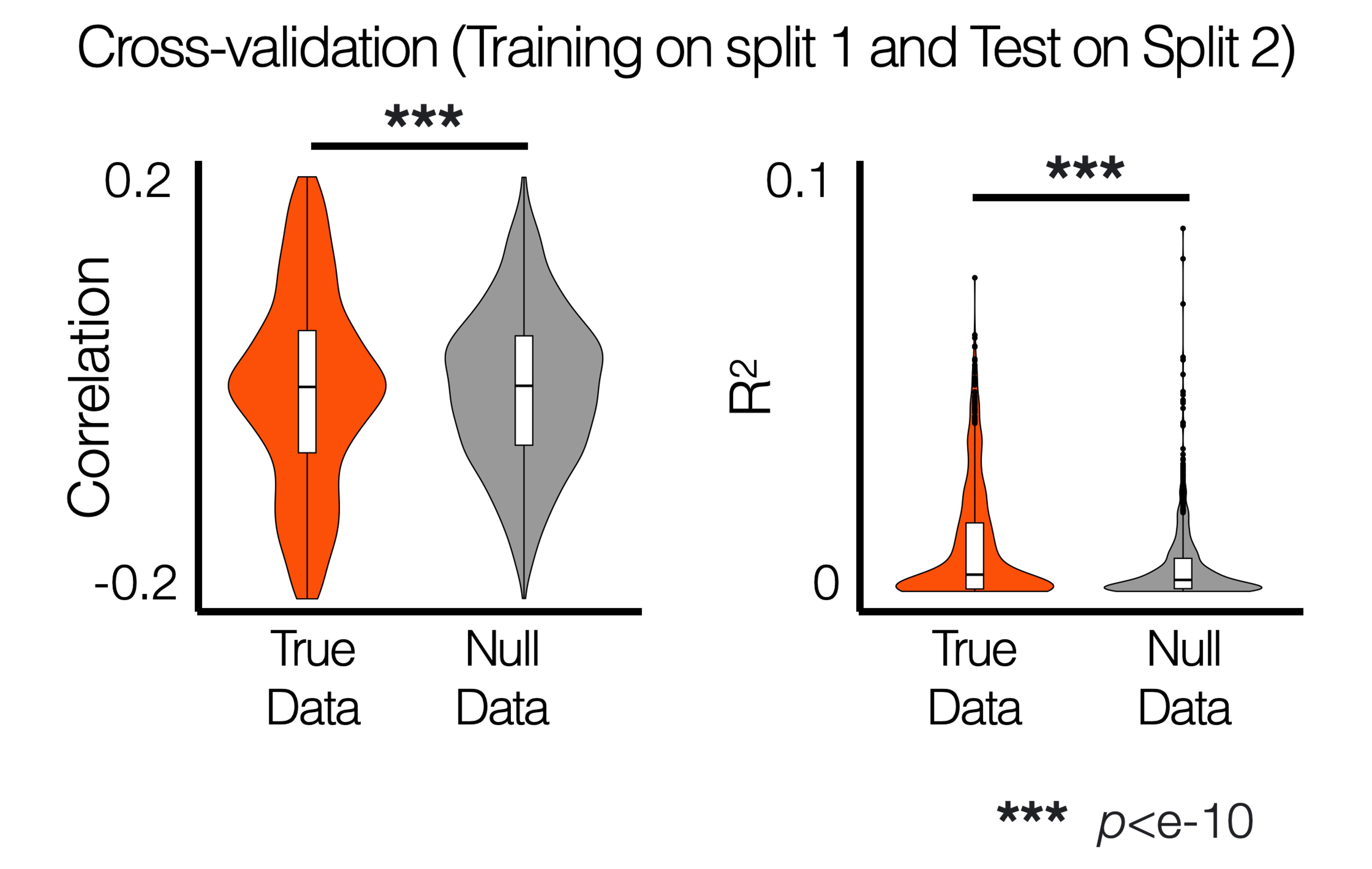

Supplement: S16 Fig — The multiple linear regression models were trained using split 1 data and tested on split 2 data in each permutation. Null data were generated by shuffling individual subjects in behavioral data. The data used to generate the results can be found in S3 Data. (TIF) [file pbio.3002808.s016.tif]

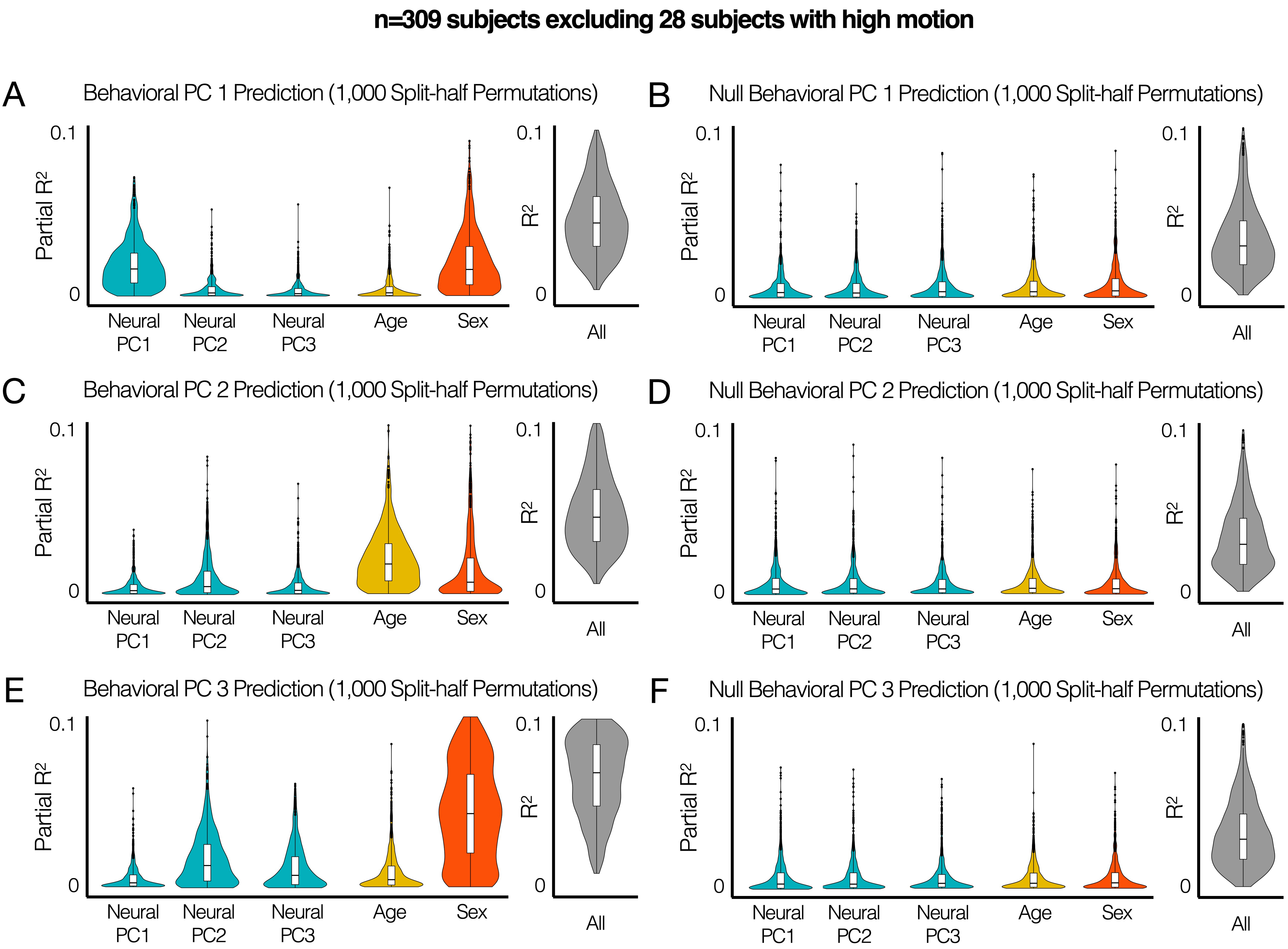

Supplement: S17 Fig — Among 337 subjects, 28 subjects with excessive motion (FD ˃ 0.5mm) were excluded. Across 1,000 permutations, a split of subjects (n = 154) was randomly selected, and PCA was performed on neural measures from these subjects. Multiple linear regression models for predicting behavioral PCs from these subjects were estimated. Null data were generated by shuffling individual subjects in behavioral data. The data used to generate the results can be found in S5 Data. (TIF) [file pbio.3002808.s017.tif]
