# Supplementary material for: Human brain state dynamics are highly reproducible and associated with neural and behavioral features
Source: PLoS Biol. 2024 Sep 24;22(9):e3002808. doi: 10.1371/journal.pbio.3002808 (PMC11421804; doi:10.1371/journal.pbio.3002808)
Supplement: Attachment — Submitted filename: CAP_PLOS_Biology_Response_to_Reviewers_final.pdf [file pbio.3002808.s023.pdf]

Response to Reviewers

Ms. No.: PBIOLOGY-D-23-02679R1
Title: Human Brain State Dynamics Reflect Neuro-Phenotypes
Corresponding Authors: Dr. Kangjoo Lee and Dr. Alan Anticevic
Authors: Dr. Kangjoo Lee, Dr. Jie Lisa Ji, Dr. Clara Fonteneau, Dr. Lucie Berkovitch, Dr. Masih Rahmati, Lining Pan, Dr. Grega Repovš, Dr. John H. Krystal, Dr. John D. Murray, Dr. Alan Anticevic

Dear editors and reviewers:

We would like to thank the editors for the opportunity to revise and resubmit our paper and the reviewers for their constructive and helpful comments. The questions raised by the reviewer are addressed below. Comments from the reviewer and any unchanged texts in the revised manuscript are written in "**black**". Our responses to comments are written in "**blue**" and any changes in the revised manuscript are highlighted in "**red**". To meet the criteria of manuscript organization for PLOS Biology, we changed the name of the section "Methods" to "Materials and Methods" and removed the "Conclusion" section, while the conclusion paragraph has been combined into the last paragraph of "Discussion" section. Please note that the page numbers (for example, (p. 3)) are provided based on the revised manuscript in the PLOS Biology format. We hope the reviewers and editors find the revised manuscript satisfactory.

Response to Reviewer #1

Comment 1. Were the subjects unrelated? If yes/no are CAPs more similar between MZ vs DZ twins?

Response: We mentioned in the manuscript that **Introduction.** "To address this, we studied rs-fMRI and behavioral data obtained from 337 unrelated healthy young adults in the individual Human Connectome Project (HCP) S1200 data." and in **Methods**. "We selected participants from the S1200 release of the HCP who had no family relations, resulting in a total of 337 participants included in our analyses." For better visibility, we revised this sentence in Introduction as follows.

(p. 3) **Introduction.** "To address this, we studied rs-fMRI and behavioral data obtained from 337 healthy young adults with no family relation in the individual Human Connectome Project (HCP) S1200 data."

We then discussed this in our discussion as follows.

(p. 19) Discussion. "Finally, an important consideration here is that we did not evaluate the impact of sample size on the estimation of CAPs and their properties. It is unknown if the low-dimensional feature set that captures a general motif of individual variation found in this study is particular for the samples in the HCP S1200 dataset or generalizable to larger and more diverse samples of the general population. In datasets with dense sampling over days, months or years, we may find another dimension of within-subject (state) variation that contributes to the general motifs of individual differences. It is possible that with a smaller sample size or different composition of the sample, there might be a reduced chance of observing a specific CAP (e.g. CAP III) or even detect new CAPs. This could occur because a particular CAP may be rare, especially when it relates to a neural pattern that is uncommon in the general population, which may be the case for neuropsychiatric or neurological symptoms. Another important aspect to consider is the extension of this work to pediatric and adolescent samples, given that there may be a substantially different configuration of CAPs as the human brain develops. Besides, in the present study, we focused on examining the patterns of CAPs in healthy young participants with no family relation. Using other dynamic functional connectivity approaches (e.g. Hidden Markov Model), it has been suggested that the FO of brain states are subject-specific and highly heritable [24]. Exploring the full HCP S1200 dataset in future work may help to address these questions and whether these CAPs and related neural measures are heritable and more similar between monozygotic or dizygotic twins."

Comment 2. Is the visualisation in 3B correct? I.e. very similar profiles between day 1 split 1 and 2 and day 2 split 1 and 2 but not between?

Response: The observed between-day variability and high reproducibility between splits within each day are one of the key findings. First of all, we do not expect to see large differences between splits 1 and 2 across permutations, as we focus on identifying a "general" motif of individual differences in neural co-activation and

evaluating how individuals occupy (or project onto) this general motif. On the other hand, we found that neural features show notable day-to-day variability (**Fig 3**), as pointed out by the Reviewer 1. The observed day-to-day variability is also evident in our 3-dimensional visualization of brain trait-state space (**Fig. 3**) and in the neural PC 3 (**Fig. 4F**). Together, we consider the observed day-to-day variability in CAP features as state variance. These results demonstrate that state variance contributes to the individual differences, therefore reflecting neuro-phenotypes. We clarified it in the text as follows.

(p.7) Results. "In this study, we are interested in testing the hypothesis that there is a reproducible general motif of individual differences in neural co-activation dynamics, where individuals differently occupy (or project onto). While previous work in [26] focused on a low-dimensional manifold of spatiotemporal neural activity by applying principal component analysis of rs-fMRI signal volumes, we aim to identify a low-dimensional feature space that characterizes state and trait properties of the temporal organizations of brain states. To do this, we first demonstrate that state-trait CAP dynamics are reproducible at the single subject level across permutations, whereas within-subject between-day reliability was lower than between-permutation reliability on a same day (**Fig. 3B, C**, **S10 Fig**)"

(p.8) Results. "Within-subject variance of FO across 5 CAPs are shown in **S12 Fig** across permutations. Together, these results show day-to-day variability (state) in CAP dynamics within individuals and highly reproducible between-subject (trait) variability within each day."

(p.10) Results. "Together, our results demonstrate that both state and trait variance of spatio-temporal CAP dynamics involve pivotal information for identifying individual differences. Specifically, we identified three neural PCs that establish a low dimensional, general motif of state and trait neural co-activation variation. The third principal component of individual variation involved information about day-to-day variability in neural co-activation, suggesting that patterns of within-subject variations can be uniquely individualized. This can be, in turn, considered as trait-like patterns providing additional information about individual neuro-phenotypes. While trait variations (neural PC 1 and 2) are dominantly loaded on the general motif of individual differences, the observed state variance at the time scale of days (neural PC 3) also contributes to this low dimensional feature space, therefore reflecting neuro-phenotypes. The assessment of individual distributions of each neural measure supported these findings (**S13 Fig**). In addition, we found that the FO of CAPs I and II have overall a higher mean and variability than the FO of CAP III. We observed the same patterns in the mean DT and var DT **S13 Fig**). Indeed, our analyses combining the hierarchical clustering and PCA of individual neural feature sets revealed three subgroups exhibiting distinct patterns of neural variations."

Comment 3. Overall the manuscript moves towards more general patterns at the end of the result. While it is interesting, I did wonder a little what then these reduced dimensions represent and whether these CAP / behavioral features are particular for HCP? (just discuss)

Response: Indeed, the low-dimensional feature space that index state and trait CAP dynamics is the discovery we want to highlight in this work. We discussed this to better emphasize the implications and importance of this finding as follows.

(p. 14) Discussion. "This study provides evidence to highlight the importance of quantifying both within-subject and between-subject variance components of brain dynamics and their link to individual differences in functional behavioral outcomes. Here, we show that the dynamics of rs-fMRI can be quantified via CAP analyses and reveal reproducible neural features that can maximize effects of state variance, trait variance, and test-retest reliability. We found that the human brain manifests a highly reproducible low-dimensional set of features that index brain-wide co-activation patterns. The neural feature reduction captures a general motif of individual variation, such that individuals occupy this low-dimensional state-trait neural space differently, which in turn predicts life and behavioral outcomes. State variance indexed by day-to-day variability in co-activation dynamics are loaded on this low-dimensional motif of individual variation, therefore reflecting neuro-phenotypes."

(p. 19) Discussion. "Finally, an important consideration here is that we did not evaluate the impact of sample size on the estimation of CAPs and their properties. It is unknown if the low-dimensional feature set that captures a general motif of individual variation found in this study is particular for the samples in the HCP S1200 dataset or generalizable to larger and more diverse samples of the general population. In datasets with dense sampling over days, months or years, we may find another dimension of within-subject (state) variation that contributes to the general motifs of individual differences. It is possible that with a smaller sample size or different composition of the sample, there might be a reduced chance of observing a specific CAP (e.g. CAP III) or even detect new CAPs. This could occur because a particular CAP may be rare, especially when it relates to a neural pattern that is uncommon in the general population, which may be the case for neuropsychiatric or neurological symptoms. Another important aspect to consider is the extension of this work to pediatric and adolescent samples, given that there may be a substantially different configuration of CAPs as the human brain develops. Besides, in the present study, we focused on examining the patterns of CAPs in healthy young participants with no family relation. Using other dynamic functional connectivity approaches (e.g. Hidden Markov Model), it has been suggested that the FO of brain states are subject-specific and highly heritable [24]. Exploring the full HCP S1200 dataset in future work may help to address these questions and whether these CAPs and related neural measures are heritable and more similar between monozygotic or dizygotic twins."

Comment 4. The authors include the full BOLD signal range, did they also test for the impact of this decision by focussing on different frequency ranges?

Response: We agree that this is an important point to be studied extensively. We did not specifically test this in this work because this requires a comprehensive analysis and we plan to do this in our future work. We discussed this as follows.

(p. 15) Discussion. "It was beyond the scope of the present study to assess the impact of temporal sampling of BOLD signals on CAP analysis. However, previous research have suggested that focusing only on particular time points, such as those during events with high BOLD signal amplitude or strong signal correlation with a seed region, while disregarding the remaining of the data (e.g. event-absent), may potentially result in misleading conclusions [44]. Iraji et al [44] noted that event-absent time points could capture a unique and robust relationship between the default mode functional network connectivity and schizophrenia symptoms. Therefore, future investigations addressing this specific question will be helpful for determining the extent to which transient brain co-activation patterns can capture diverse characteristics of individual brain dynamics. For

instance, CAP derived measures can be quantified from data with various temporal sampling strategies, such as varying the proportion of analyzed time points randomly or based on specific signal criteria. In addition, the impacts of temporal resolution of fMRI BOLD acquisition (e.g. time or repetition; TR) should be given further consideration."

Comment 5. In the introduction the CAP framework as framed as a compromise between state and trait-like patterns in the brain, but in the final figures CAPs are further put into a trait-like perspective, also linking to behaviours. How to interpret this?

Response: We understand that this interpretation was unclear and revised the manuscript to better clarify our key argument as follows. As we mentioned in our responses to your Comments 6, we argue that the observed day-to-day variability in CAP dynamics reflects state-related features, which in turn can provide information about individual neuro-phenotypes - in other words, trait-like patterns. By identifying three neural PCs that together establish a low dimensional space of state and trait neural co-activation variation (particularly neural PC 3 representing day-to-day variability), we show that patterns of state variations can be also individual specific that can be, in turn, considered as traits. In this context, state variation associated with day-to-day variation in CAP profiles can play a role in an individual's neuro-phenotype. This key finding is also reflected in the title of this manuscript "Human brain state dynamics reflect individual neuro-phenotypes".

(p.10) Results. "Together, our results demonstrate that both state and trait variance of spatio-temporal CAP dynamics involve pivotal information for identifying individual differences. Specifically, we identified three neural PCs that establish a low dimensional, general motif of state and trait neural co-activation variation. The third principal component of individual variation involved information about day-to-day variability in neural co-activation, suggesting that patterns of within-subject variations can be uniquely individualized. This can be, in turn, considered as trait-like patterns providing additional information about individual neuro-phenotypes. While trait variations (neural PC 1 and 2) are dominantly loaded on the general motif of individual differences, the observed state variance at the time scale of days (neural PC 3) also contributes to this low dimensional feature space, therefore reflecting neuro-phenotypes.} The assessment of individual distributions of each neural measure supported these findings (**S13 Fig**). In addition, we found that the FO of CAPs I and II have overall a higher mean and variability than the FO of CAP III. We observed the same patterns in the mean DT and var DT (**S13 Fig**). Indeed, our analyses combining the hierarchical clustering and PCA of individual neural feature sets revealed three subgroups exhibiting distinct patterns of neural variations."

Comment 6. The concept of reproducibility seems core to the hypothesis of the work, yet ICC for individuals is fairly low, what would this mean for the framework? Does this also relate to the state-trait conundrum?

Response: Thank you for the insightful comment. We have added our interpretation in Discussion as follows.

(p.16) Discussion. "Collectively, these results highlight that state-trait CAP dynamics are reproducible at the single subject level across permutations and splits (**Fig. 3, S9 Fig**). For context, the statistics reported here (**Fig. 3**C) demonstrate higher reproducibility than the meta-analytic estimate for group-level reproducibility of area-to-area functional connectivity matrices [51]. Still, the observed ICC values are fairly low, reflecting a notable

variance between days. This indicates that while trait-like features are most dominant factors loaded on the general motif of individual variance, the observed state variance (between days) also contributes to this general motif. In this context, the fairly low range of ICC reflects the notable amount of within-subject variance at the time-scale of days, and supports our framework of state-trait components that together identify neural phenotypes. Similarly, Yang et al. found that CAPs identified at individual level were unstable over time across the ten scans (~ 30 minutes/scan) except a few subjects, and subject-specific CAPs became more reliable and individual specific when integrating data with longer duration [55]. Therefore, we argue that such temporal fluctuations within subjects can bring critical insights into individual-specific brain organizations. Reducing the number of neural features into a reproducible set of CAPs may enable a more robust and reproducible mapping between neural features and behavior. In other words, we hypothesize that further optimization of reproducible data-reduced neural features presents a critical step toward mapping rs-fMRI signals to healthy and clinically-relevant behavioral variation and obtaining robust neuro-behavioral models.**"**

Comment 7. moreover another aim is to link CAP to behavioral phenotypes across multiple behavioral domains, yet in the end only the principle component is selected?

Response: It was not our goal to study neuro-behavioral association in individual behavioral variables or domains separately, because that information in both neural and behavioral data involve high dimensional complexity. Rather, we aimed at (1) identifying a low-dimensional neural feature space that best characterizes individual differences in brain dynamics, and (2) studying to what extent individual variability quantified on this neural feature space can be linked to patterns of individual human behavior. To achieve this, we employed a dimension reduction strategy to estimate the behavioral PCs that provide low-dimensional geometries across multiple behavioral domains, where people occupy differently. This way, we can associate how individual subjects are distributed in two feature spaces that are estimated from neural data and behavioral data respectively, and how the patterns relate to each other. We mentioned this in the Results section as follows.

(p. 10) Results. "**Principal variations of neural state-trait features co-vary with principal variations of behavioral phenotypes.** Next, we were interested in studying to what extent individual variability quantified on this low-dimensional neural feature space was linked to variations of individual human behavior. We employed a similar dimension reduction strategy to estimate the behavioral principal components (PCs) that provide low-dimensional geometries across multiple behavioral domains where people occupy differently. This way, we can associate how individual subjects are distributed in two feature spaces respectively and how such patterns relate to each other."

To enrich our analysis of neuro-behavioral mapping on the low-dimensional space, we extended our analysis to associate the first three neural PCs and the first three behavioral PCs. We ensured the reproducibility of our neuro-behavioral mapping using permutation and cross-validation, and we tested the impact of motion. Figure 6, Supplementary Figures S15, S16 and S17 were added as shown below.

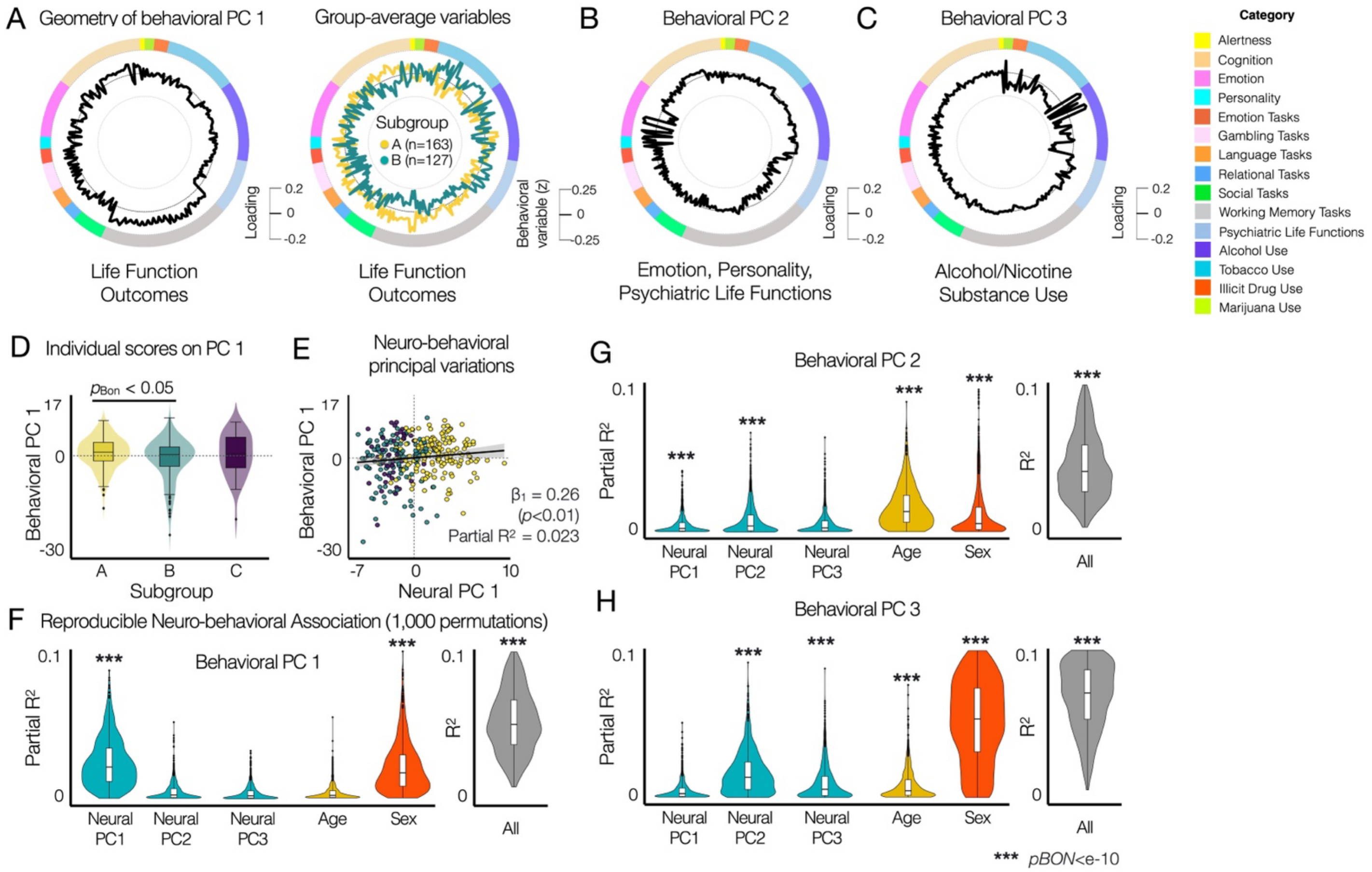

Figure 6. "**Principal variations of neural state-trait features co-vary with the principal variations of behavioral phenotypes, highlighting individual life function outcomes associated with emotion regulation, cognitive function and alcohol and substance use.** **(A)** The geometry of behavioral PC 1 (black, left circle) reflects the difference in group-average behavioral variables (standardized behavioral data, right circle) between subgroups **A** (yellow) and **B** (green). Subgroup **C** is not shown because no significant group differences are found in (D). **(B)** The geometry of behavioral PC 2. **(C)** The geometry of behavioral PC 3. **(D)** Comparison of individual PC 1 scores between subgroups identified using neural state-trait measures (Fig. 4). Two-sample two-sided t-tests were performed between subgroups for each behavioral PC. p_{BON}: Bonferroni corrected p-values. **(E)** Multiple linear regression model of three neural PC 1 with two covariates (age and sex) showed that the neural PC 1 was associated with the behavioral PC 1 (Partial $R^2 = 0.023$, $\beta_1 = 0.26$, SE = 0.09, t = 2.8, p = 0.006), where multiple $R^2 = 0.041$, adjusted $R^2 = 0.026$, $F(5, 331) = 2.814$, p-value = 0.017 for the full model. **(F-H)** Reproducibility analysis of the prediction of individual behavioral PC scores from neural PCs. In each permutation, PCA was performed for the neural and behavioral data from subjects in a random half of the entire sample (N=168). Parameters of multiple linear regression models with three neural PC 1 with two covariates (age and sex) were estimated to evaluate the predictability of each behavioral PC. p_{BON}: Bonferroni corrected p-values from F-tests."

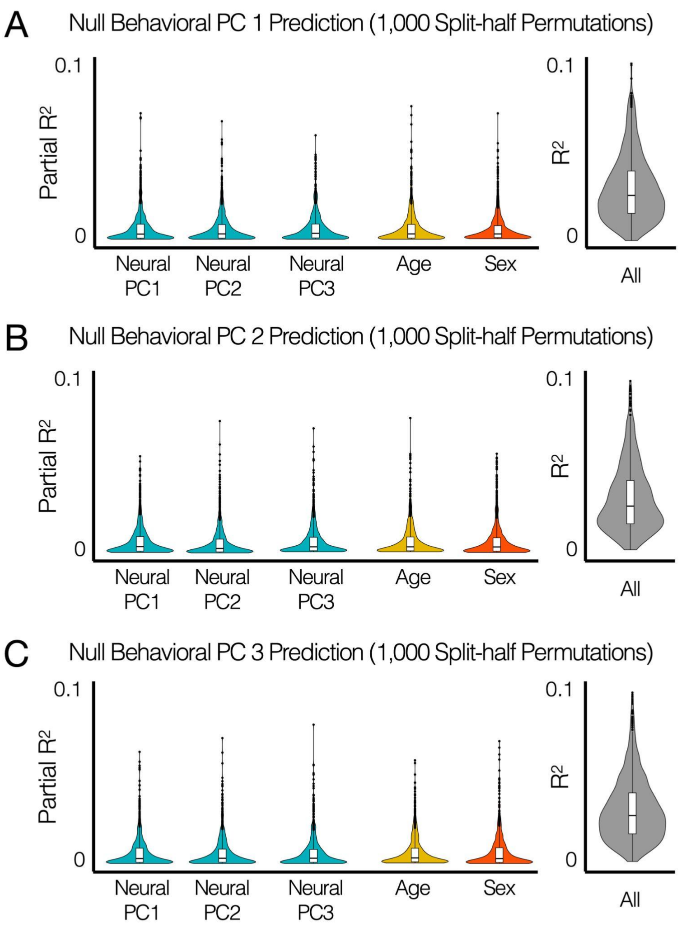

S15 Fig. Null data were generated by shuffling individual subjects in behavioral data. Null distributions of partial R^2 were estimated for each predictor in the neuro-behavioral association model trained from a split data across 1,000 split-half permutations."

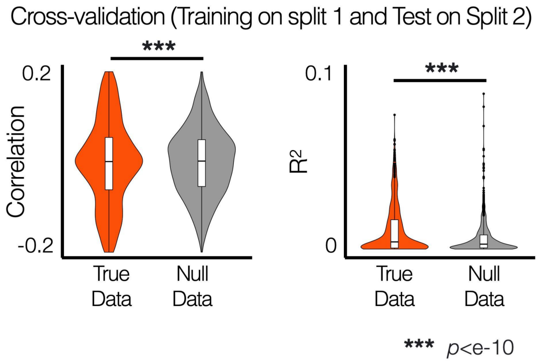

S16 Fig. Split-half permutation based cross-validation of the prediction model of predicting behavioral PC 1 from neural PCs. The multiple linear regression models were trained using split 1 data and tested on split 2 data in each permutation. Null data were generated by shuffling individual subjects in behavioral data."

(p. 13) Results. "**Reproducibility and Cross-validation of Low-Dimensional Neuro-Behavioral Association.**

To further evaluate the reproducibility of the neuro-behavioral association in our low-dimensional space found in **Fig. 6E**, we first performed the same multiple linear regression approach on a split data (random N=168)

across 1,000 permutations. Null data were generated by shuffling individual subjects in each behavioral PC data (**S15 Fig**). For predicting behavioral PC 1, the resulting partial R^2 values were strongly reproducible across permutations (partial R^2 =0.025 ± 0.017 for neural PC1, p_{BON} <e-10, F-test; overall R^2=0.056 ± 0.024), as shown in **Fig. 6F**. Similar to the analysis using the entire data, sex was also a reproducible predictor of behavioral PC 1 (partial R^2 =0.02 ± 0.016, p_{BON} <e-10, F-test). Secondly, we used the multiple linear regression model trained from each split 1 data for predicting individual behavioral scores in the corresponding split 2 data across permutations (**S16 Fig**). While the overall prediction performance was relatively low, it was highly reproducible and significantly different from null data analysis (R^2=0.011 ± 0.013, p<e-10, F-test).

Next, we repeated the same analyses for predicting behavioral PCs 2 and 3. Neural PCs 1 and 2 showed reproducible association with behavioral PC 2 that highlights emotion, personality, and psychiatric life functions, whereas age and sex showed larger predictive power (**Fig. 6G**). For predicting behavioral PC 2, the estimated partial R^2 was 0.008 ± 0.01 for neural PC2, 0.018 ± 0.014 for age, and 0.013 ± 0.019 for sex (**Fig. 6G**). On the other hand, neural PC 2 was predictive of behavioral PC 3 highlighting a strong association with alcohol consumption habits and sex differences (**Fig. 6H**). For predicting behavioral PC 2, the estimated partial R^2 was 0.016 ± 0.014 for neural PC2 and 0.059 ± 0.036 for sex, whereas the overall R^2 for the full model was 0.098 ± 0.041 (**Fig. 6H**)."

(p. 13) Results. "**Impact of motion.** To evaluate the impact of motion, the mean Frame Displacement (FD) was computed across time-frames for each subject. The estimated mean FD was 0.16 +/- 0.58 across subjects (N=337) . Note that we scrubbed time-frames with excessive motion (FD>0.5 mm) when estimating CAPs (**S1 Fig**). The average number of scrubbed time-frames across subjects was 76 +/- 192.6 (counts), which are 1.73 +/- 4.38 % of the total number of time-frames (4,400/subject before removing dummy scans) in each subject. More than 5% of total time-frames were scrubbed in 28/337 subjects (8.3%). We also measured the duration of motion (the number of consecutive time-frames with excessive motion) to assess whether there were long time-segments of motion, which might impact the estimation of temporal CAP profiles, especially analyses of CAP state transitions and dwell time. The length of motion-related continuous time-frames was 1.34 +/-0.38 time-frames on average across subjects. Repeating the analyses excluding these 28 subjects did not change the results, including the PCA of neural measures and the low-dimensional neuro-behavioral relationships (**S17 Fig**).

In summary, we tested the hypothesis that there is a reproducible CAP feature set that reflects both state and trait brain dynamics and that this combined feature set relates to individual phenotypes across multiple behavioral domains. Specifically, **behavioral PC 1** highlights individual life function outcomes associated with cognition, emotion regulation, alcohol use and substance use. Individuals with high behavioral PC 1 scores are found to (i) spend longer time at CAP I than at CAP II, (ii) have higher between-subject variance and lower within-subject variance at CAP I than at CAP II, (iii) show high global persistence (longer dwell time) in all CAPs, and (iv) lower chance to have CAP III. Such neuro-behavioral patterns were (v) associated with sex differences. **Behavioral PC 2** highlights emotion regulation, personality and psychiatric life functions. Individuals with high behavioral PC 2 scores are found to (i) show longer dwell time in all CAPs, (ii) spend longer time at CAP I than at CAP II, and (iii) be associated with Age. **Behavioral PC 3** highlights alcohol, nicotine and substance use. Individuals with high behavioral PC 3 scores are found to (i) have longer dwell time in all CAPs, (ii) higher chance to have CAP III, and (iii) be associated with sex differences. "

Comment 8. To what extend does the spatial alignment of co-activation patterns in individuals contributes to the observed cap variability?
(correlation between individual CAP frames to basis CAPs – dist of correlation)

Response: Thank you for this interesting question. Because the spatial CAPs are estimated from a concatenated data matrix of fMRI time-frames obtained from 2/N subjects, we consider "co-activation patterns in individuals" in your comment as "the fMRI time-frames in individuals". We investigated the correlation between individual time-frames to the basis CAPs and addressed this question as follows.

(p.6) **Results. "Spatial alignment of individual time-frames to basis CAPs.** To find an optimal number of clusters or CAPs that are commonly found across individuals, we used an approach that considers a trade-off between the number of clusters and within-cluster similarity by combining the silhouette criteria and elbow method (**S2 Fig**). To evaluate the extent of the contribution of individual co-activation patterns to the observed CAP variability, we analyzed all fMRI time-frames obtained from 337 subjects after scrubbing. For each split, we computed the spatial alignment of individual 3-dimensional fMRI time-frames to the five basis CAPs (cluster centroids estimated by K-means clustering) using Pearson's correlation, identifying a basis CAP yielding the highest correlation with each time-frame. As a result, the mean and standard deviation of the maximum correlation were 0.22 ± 0.11 (**S8 Fig**), indicating the substantial variability in resting state human brain dynamics. Notably, the group-level spatial topography of CAPs, estimated by averaging the time-frames within each cluster, remained consistent across permutations (**Fig. 2**), enabling us to investigate individual differences in their temporal dynamics.**"**

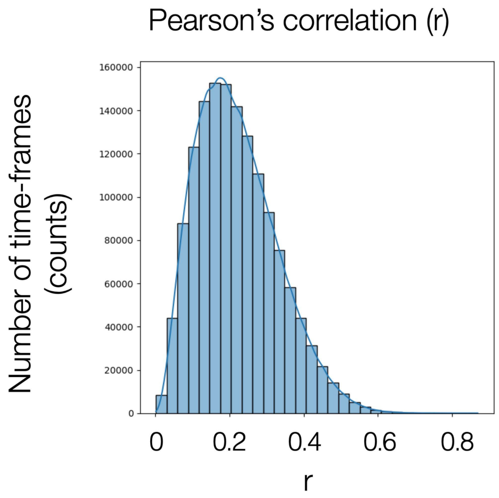

S8 Fig. The distribution of correlations between individual fMRI time-frames and the estimated basis CAPs (cluster centroid), to which individual time-frames were assigned by K-means clustering.

Response to Reviewer #2

Comment 1. The authors emphasize the use of CAPs (i.e., low-dimensional brain states) in characterizing state-trait variance. Though the authors review past CAPs literature on patient population, the authors omitted quite many seminal studies that identified latent brain states—including features such as fractional occupancy, dwell times, etc—and related with state and trait variances. I listed some of the work, just to name a few; Vidaurre et al. (2017) even used the same HCP trait measures. I agree that the majority of work (especially initial work) focused on trait-level neural characteristics to relate with individual phenotypes (e.g., Finn et al., 2015); however, using neural state dynamics or time-varying functional connectivity to understand individual differences is also an active area of research. Please discuss the current manuscript in relation to past literature.
Vidaurre, Smith & Woolrich, 2017, PNAS (https://www.pnas.org/doi/full/10.1073/pnas.1705120114): various trait measures in HCP
Shine et al., 2019, Nat Neurosci (https://www.nature.com/articles/s41593-018-0312-0): fluid intelligence
Taghia et al., 2018, Nat Commun (https://www.nature.com/articles/s41467-018-04723-6): working memory performance

Response: We thank the reviewer for suggesting to include these important past literature. We discussed these works in the revised manuscript, as follows.

Updated References.
[24] Vidaurre, Smith & Woolrich,Brain network dynamics are hierarchically organized in time, PNAS, 2017
[25] Taghia et al., Uncovering hidden brain state dynamics that regulate performance and decision-making during cognition, Nat Communications, 2018
[26] Shine et al., Human Cognition involves the dynamic integration of neural activity and neuromodulatory systems, Nat Neuroscience 2019
[61] Larsen et al., A critical period plasticity framework for the sensorimotor-association axis of cortical development, Trends in Neuroscience, 2023
[62] Luo et al., Functional connectivity development along the sensorimotor-association axis enhances the cortical hierarchy, Nature Communications, 2024

(p. 2) Introduction. " The analyses of neural state dynamics or time-varying rs-fMRI connectivity can be used to understand individual differences [24]. Evaluating moment-to-moment changes in neural activity can provide information about latent brain states associated with task-switching and decision-making in working memory [25]. Using dimension reduction of task fMRI data across multiple cognitive tasks, Shine et al. suggested that execution of diverse cognitive tasks and individual differences in fluid intelligence can be described using a dynamic flow along a low-dimensional manifold of global brain activity [26]. There is a knowledge gap regarding how combined state and trait variation of spontaneous brain dynamics map onto individual variation in complex behavioral phenotypes."

(p.7) Results. "In this study, we are interested in testing the hypothesis that there is a reproducible general motif of individual differences in neural co-activation dynamics, where individuals differently occupy (or project

onto). While previous work in [26] focused on a low-dimensional manifold of spatiotemporal neural activity by applying principal component analysis of rs-fMRI signal volumes, we aim to identify a low-dimensional feature space that characterizes state and trait properties of the temporal organizations of brain states. To do this, we first demonstrate that state-trait CAP dynamics are reproducible at the single subject level across permutations, whereas within-subject between-day reliability was lower than between-permutation reliability on a same day (**Fig. 3B, C**, **S10 Fig**)"

(p. 16) Discussion. "Similarly, several brain states were estimated at the group level using another dynamic functional connectivity approach in [24], whereas they could identify the time-points when the state was active, allowing the estimation of FO for each state and for each subject. **"**

(p.18) Discussion. "Our results converge with several findings using other dynamic functional connectivity approaches. Vidaurre et al. used Hidden Markov Model to identify 12 brain states using the HCP-YA rs-fMRI datasets and derived two metastates, each being a set of brain states that are more likely to transit between each other [24]. They found that the FO of brain states and their associated metastates are subject-specific and behaviorally relevant, highlighting several well-being, intelligence and personality traits [24]. It would be interesting to evaluate, using the same subjects studied in this work, if brain activity during cognitive tasks exhibit a similar set of CAP features and low-dimensional state-trait variances to those estimated using rs-fMRI and how they are related to such high-order metastates [24] and the low-dimensional global brain activity found across multiple cognitive tasks [26].**"**

(p. 19) Discussion. "Our results in the reproducible neuro-behavioral associations highlighted the neural relevance of behavioral PCs 1, 2 and 3. Individuals with higher cognition (behavioral PC 1) spent longer time at CAP I than at CAP II. The behavioral PC 2 showed an association with neural PCs 1 and 2, as well as age and sex. In relation to resting state functional connectivity, While CAPs I and II show differences in the regions of language, primary visual, and cingulo-opercular networks, the overall patterns of these CAP states involved opposing patterns of brain activity in the default mode and frontoparietal networks versus the regions belonging to the secondary visual, somatomotor and dorsal attention networks [61]. This suggests a relation to the cortical hierarchy that spans from unimodal sensorimotor cortices to transmodal association cortices and a potential role in functional connectivity development during childhood through adolescence [62].

Finally, an important consideration here is that we did not evaluate the impact of sample size on the estimation of CAPs and their properties. It is unknown if the low-dimensional feature set that captures a general motif of individual variation found in this study is particular for the samples in the HCP S1200 dataset or generalizable to larger and more diverse samples of the general population. In datasets with dense sampling over days, months or years, we may find another dimension of within-subject (state) variation that contributes to the general motifs of individual differences. It is possible that with a smaller sample size or different composition of the sample, there might be a reduced chance of observing a specific CAP (e.g. CAP III) or even detect new CAPs. This could occur because a particular CAP may be rare, especially when it relates to a neural pattern that is uncommon in the general population, which may be the case for neuropsychiatric or neurological symptoms. Another important aspect to consider is the extension of this work to pediatric and adolescent samples, given that there may be a substantially different configuration of CAPs as the human brain develops. Besides, in the present study, we focused on examining the patterns of CAPs in healthy young participants with no family relation. Using other dynamic functional connectivity approaches (e.g. Hidden Markov Model), it has been

suggested that the FO of brain states are subject-specific and highly heritable [24]. Exploring the full HCP S1200 dataset in future work may help to address these questions and whether these CAPs and related neural measures are heritable and more similar between monozygotic or dizygotic twins."

Comment 2. The first sentence of the abstract needs to be reworded: "Neural activity and behavior manifest state and trait dynamics, as well as variation within and between individuals". My first impression was: aren't "state and trait dynamics" and "variation within and between individuals" the same thing? My second thought was: that variation across individuals (e.g., people have different static functional connectivity patterns) and trait dynamics (e.g., people are characterized by some recurring dynamics) could potentially be different, but how is variation within individuals and state dynamics different? Either way of interpretation, this sentence is confusing.

Response: We agree that the sentence is confusing, and revised the sentence to better clarify, as follows.

(p.1) Abstract. "Neural activity and behavior vary within an individual (states) and between individuals (traits)."

Comment 3. The main finding of this paper is Fig. 6G. Is this result robust? To test for robustness/predictability, could the authors, for example, conduct neural and behavioral PCA on split-half, fit the regression model, and apply these to the other split-half by transforming the values to PC space and testing if the regression output is predictable?

Response: Thank you for the suggestion. We first performed split-half resampling based analysis of reproducibility of the neuro-behavioral associations in low-dimensional space as follows. Figure 6 and the corresponding texts were added as shown below. We added a S15 Fig to show null data distribution for Partial R^2 values across split-half permutations. Secondly, we performed a split-half cross validation, as suggested by the reviewer, to predict individual behavioral PC 1 in split 2 data using the model trained from split 1 data (S16 Fig). We also tested the impact of motion on this analysis and confirmed that removing subjects with high motion did not change the results (S17 Fig).

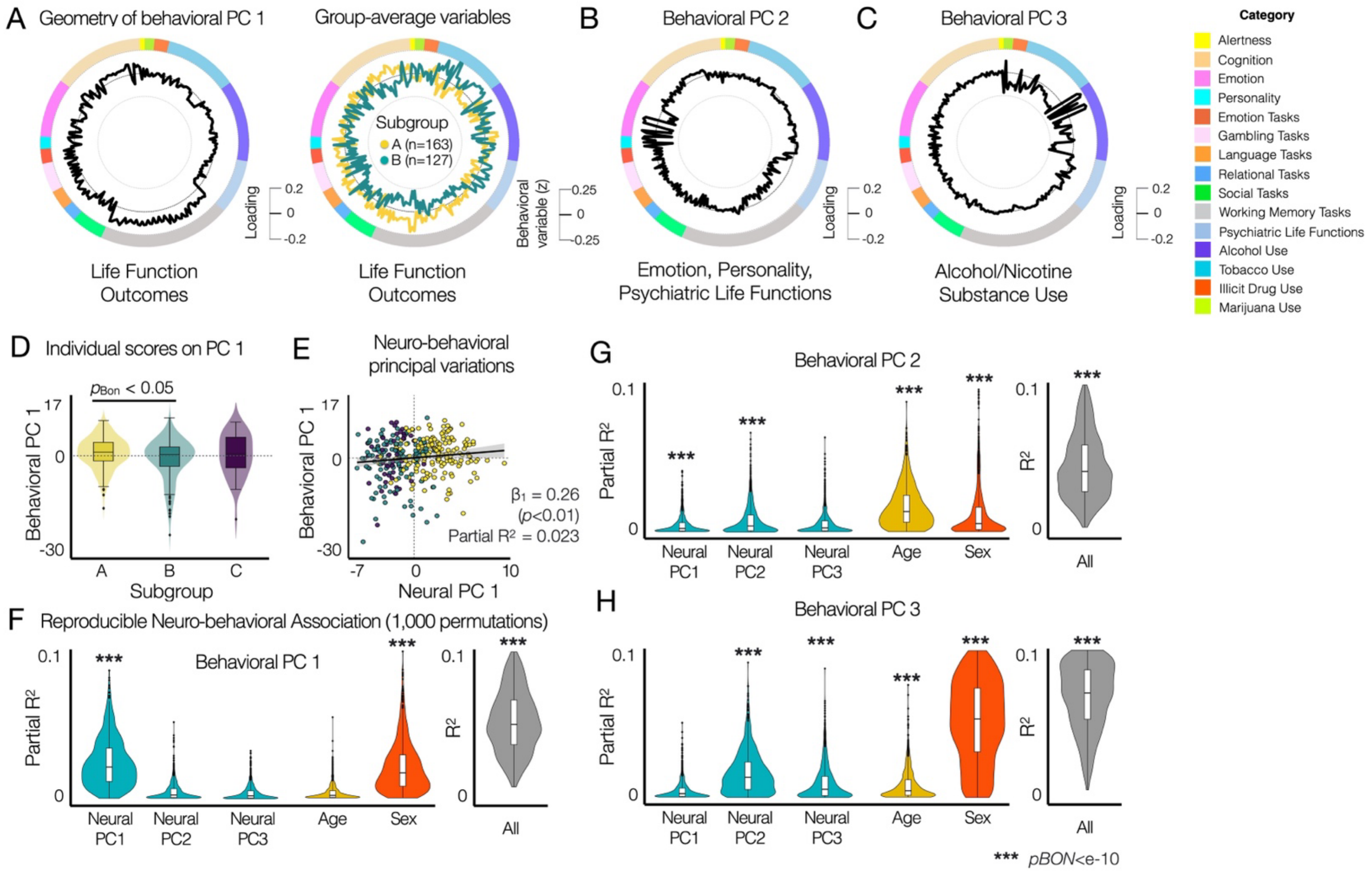

Figure 6. "**Principal variations of neural state-trait features co-vary with the principal variations of behavioral phenotypes, highlighting individual life function outcomes associated with emotion regulation, cognitive function and alcohol and substance use. (A)** The geometry of behavioral PC 1 (black, left circle) reflects the difference in group-average behavioral variables (standardized behavioral data, right circle) between subgroups **A** (yellow) and **B** (green). Subgroup **C** is not shown because no significant group differences are found in (D). **(B)** The geometry of behavioral PC 2. **(C)** The geometry of behavioral PC 3. **(D)** Comparison of individual PC 1 scores between subgroups identified using neural state-trait measures (Fig. 4). Two-sample two-sided t-tests were performed between subgroups for each behavioral PC. p_{BON}: Bonferroni corrected p-values. **(E)** Multiple linear regression model of three neural PC 1 with two covariates (age and sex) showed that the neural PC 1 was associated with the behavioral PC 1 (Partial $R^2 = 0.023$, $\beta_1 = 0.26$, SE = 0.09, t = 2.8, p = 0.006), where multiple $R^2 = 0.041$, adjusted $R^2 = 0.026$, F(5, 331)=2.814, p-value = 0.017 for the full model. **(F-H)** Reproducibility analysis of the prediction of individual behavioral PC scores from neural PCs. In each permutation, PCA was performed for the neural and behavioral data from subjects in a random half of the entire sample (N=168). Parameters of multiple linear regression models with three neural PC 1 with two covariates (age and sex) were estimated to evaluate the predictability of each behavioral PC. p_{BON}: Bonferroni corrected p-values from F-tests."

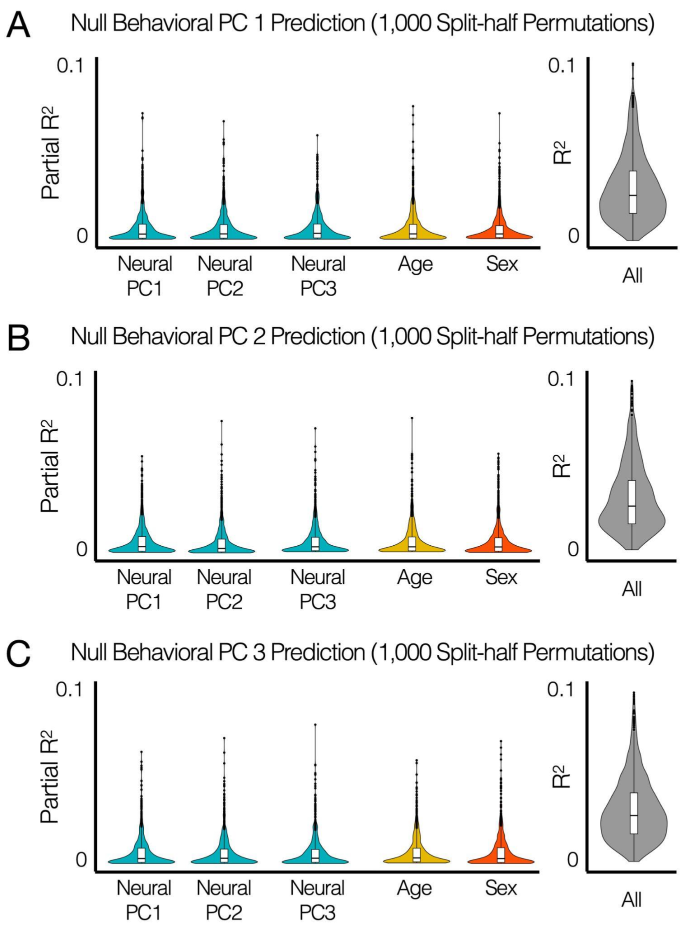

S15 Fig. Null data were generated by shuffling individual subjects in behavioral data. Null distributions of partial R^2 were estimated for each predictor in the neuro-behavioral association model trained from a split data across 1,000 split-half permutations."

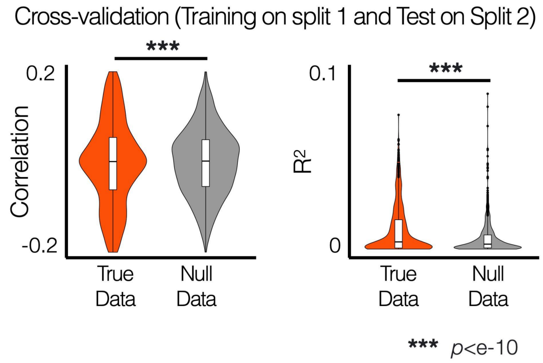

S16 Fig. Split-half permutation based cross-validation of the prediction model of predicting behavioral PC 1 from neural PCs. The multiple linear regression models were trained using split 1 data and tested on split 2 data in each permutation. Null data were generated by shuffling individual subjects in behavioral data."

(p. 13) Results. "**Reproducibility and Cross-validation of Low-Dimensional Neuro-Behavioral Association.**

To further evaluate the reproducibility of the neuro-behavioral association in our low-dimensional space found in **Fig. 6E**, we first performed the same multiple linear regression approach on a split data (random N=168)

across 1,000 permutations. Null data were generated by shuffling individual subjects in each behavioral PC data (**S15 Fig**). For predicting behavioral PC 1, the resulting partial R^2 values were strongly reproducible across permutations (partial R^2 =0.025 ± 0.017 for neural PC1, p_{BON} <e-10, F-test; overall R^2=0.056 ± 0.024), as shown in **Fig. 6F**. Similar to the analysis using the entire data, sex was also a reproducible predictor of behavioral PC 1 (partial R^2 =0.02 ± 0.016, p_{BON} <e-10, F-test). Secondly, we used the multiple linear regression model trained from each split 1 data for predicting individual behavioral scores in the corresponding split 2 data across permutations (**S16 Fig**). While the overall prediction performance was relatively low, it was highly reproducible and significantly different from null data analysis (R^2=0.011 ± 0.013, p<e-10, F-test).

Next, we repeated the same analyses for predicting behavioral PCs 2 and 3. Neural PCs 1 and 2 showed reproducible association with behavioral PC 2 that highlights emotion, personality, and psychiatric life functions, whereas age and sex showed larger predictive power (**Fig. 6G**). For predicting behavioral PC 2, the estimated partial R^2 was 0.008 ± 0.01 for neural PC2, 0.018 ± 0.014 for age, and 0.013 ± 0.019 for sex (**Fig. 6G**). On the other hand, neural PC 2 was predictive of behavioral PC 3 highlighting a strong association with alcohol consumption habits and sex differences (**Fig. 6H**). For predicting behavioral PC 2, the estimated partial R^2 was 0.016 ± 0.014 for neural PC2 and 0.059 ± 0.036 for sex, whereas the overall R^2 for the full model was 0.098 ± 0.041 (**Fig. 6H**)."

(p. 13) Results. "**Impact of motion.** To evaluate the impact of motion, the mean Frame Displacement (FD) was computed across time-frames for each subject. The estimated mean FD was 0.16 +/- 0.58 across subjects (N=337) . Note that we scrubbed time-frames with excessive motion (FD>0.5 mm) when estimating CAPs (**S1 Fig**). The average number of scrubbed time-frames across subjects was 76 +/- 192.6 (counts), which are 1.73 +/- 4.38 % of the total number of time-frames (4,400/subject before removing dummy scans) in each subject. More than 5% of total time-frames were scrubbed in 28/337 subjects (8.3%). We also measured the duration of motion (the number of consecutive time-frames with excessive motion) to assess whether there were long time-segments of motion, which might impact the estimation of temporal CAP profiles, especially analyses of CAP state transitions and dwell time. The length of motion-related continuous time-frames was 1.34 +/-0.38 time-frames on average across subjects. Repeating the analyses excluding these 28 subjects did not change the results, including the PCA of neural measures and the low-dimensional neuro-behavioral relationships (**S17 Fig**).

In summary, we tested the hypothesis that there is a reproducible CAP feature set that reflects both state and trait brain dynamics and that this combined feature set relates to individual phenotypes across multiple behavioral domains. Specifically, **behavioral PC 1** highlights individual life function outcomes associated with cognition, emotion regulation, alcohol use and substance use. Individuals with high behavioral PC 1 scores are found to (i) spend longer time at CAP I than at CAP II, (ii) have higher between-subject variance and lower within-subject variance at CAP I than at CAP II, (iii) show high global persistence (longer dwell time) in all CAPs, and (iv) lower chance to have CAP III. Such neuro-behavioral patterns were (v) associated with sex differences. **Behavioral PC 2** highlights emotion regulation, personality and psychiatric life functions. Individuals with high behavioral PC 2 scores are found to (i) show longer dwell time in all CAPs, (ii) spend longer time at CAP I than at CAP II, and (iii) be associated with Age. **Behavioral PC 3** highlights alcohol, nicotine and substance use. Individuals with high behavioral PC 3 scores are found to (i) have longer dwell time in all CAPs, (ii) higher chance to have CAP III, and (iii) be associated with sex differences. "

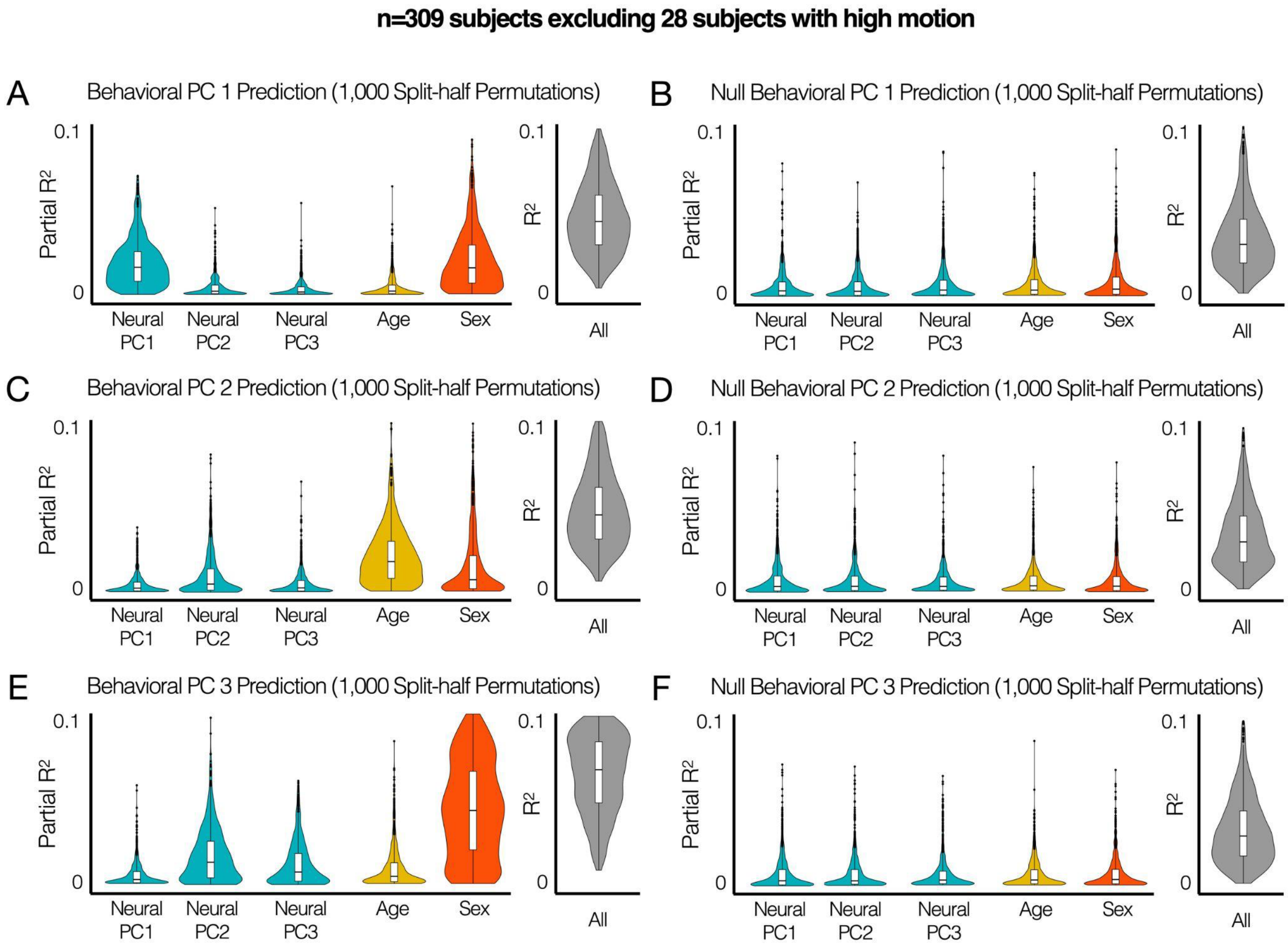

S17 Fig. Repeating the analysis of neuro-behavioral association excluding subjects with high motion did not change the results. Among 337 subjects, 28 subjects with excessive motion (FD>0.5 mm) were excluded. Across 1,000 permutations, a split of subjects (n=154) was randomly selected, and PCA was performed on neural measures from these subjects. Multiple linear regression models for predicting behavioral PCs from these subjects were estimated. Null data were generated by shuffling individual subjects in behavioral data.

Comment 4. The authors argue that the study "demonstrate a reproducible estimation of spatio-temporal CAP features at the single-subject level". However, there is a nuance - whether the results are truly at the "single-subject level". The basis set of CAPs needed to be estimated on the concatenated group time series. I do not know to what extent CAPs generated at the single-subject level are comparable to the basis set of CAPs. The features (e.g., FO, mean DT, var DT, across-day reliability) may be generated at individual subject level, but because the model was built from the aggregated group data from the start, I am unsure to what extent this sentence is warranted.

Response: We understand that the notion of "demonstrate a reproducible estimation of spatio-temporal CAP features at the single-subject level" is inaccurate and can be misleading. In our work, the spatial topography of CAPs are defined at the group level, whereas the temporal characteristics of these CAPs were quantified at the single subject level to allow the quantification of state and trait dynamics for each subject. Then, we

demonstrated the reproducibility of these temporal characteristics at the single subject level. We revised this sentence as follows.

(p.19) **Conclusions.** "Critically, we demonstrate a reproducible estimation of spatial CAP features at the group level and the temporal characteristics of CAP states at the single-subject level."

In addition, we investigated the association of spatial variability of individual co-activation patterns (single time-frames) to the group level basis CAPs., as shown below.

(p.6) **Results.** "**Spatial alignment of individual time-frames to basis CAPs.** To find an optimal number of clusters or CAPs that are commonly found across individuals, we used an approach that considers a trade-off between the number of clusters and within-cluster similarity by combining the silhouette criteria and elbow method (**S2 Fig**). To evaluate the extent of the contribution of individual co-activation patterns to the observed CAP variability, we analyzed all fMRI time-frames obtained from 337 subjects after scrubbing. For each split, we computed the spatial alignment of individual 3-dimensional fMRI time-frames to the five basis CAPs (cluster centroids estimated by K-means clustering) using Pearson's correlation, identifying a basis CAP yielding the highest correlation with each time-frame. As a result, the mean and standard deviation of the maximum correlation were 0.22 ± 0.11 (**S8 Fig**), indicating the substantial variability in resting state human brain dynamics. Notably, the group-level spatial topography of CAPs, estimated by averaging the time-frames within each cluster, remained consistent across permutations (**Fig. 2**), enabling us to investigate individual differences in their temporal dynamics."

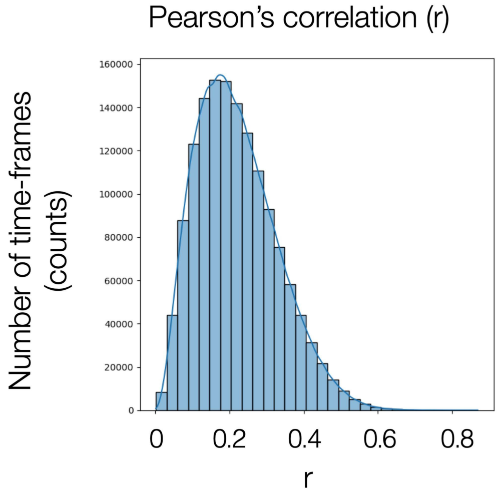

S8 Fig. The distribution of correlations between individual fMRI time-frames and the estimated basis CAPs (cluster centroid), to which individual time-frames were assigned by K-means clustering.

Our observation in S8 Fig suggests that individual co-activation time-frames involve a substantial amount of variability. Traditionally, the estimation and analysis of CAPs have been implemented at the group level, and the field is lacking a robust framework to quantify reproducible spatial patterns of individual co-activation and derive an interpretable and reproducible neuro-behavioral relationship. A recent work by Yang et al., (2023) suggests a method to detect individual CAPs at the subject-level by maximizing individual identifiability by measuring the gap between within-subject and between-subject similarity. We discussed this in the revised manuscript as follows.

(p. 16) Discussion. "A recent work suggests a method to detect individual CAPs at the subject-level by maximizing individual identifiability [55]. Using the densely sampled Midnight Scan Club dataset [56], they identified four CAPs at both the group-level and single-subject level, with two CAP pairs exhibiting opposing spatial patterns, similar to the findings in this work (**Fig. 1**). However, their study did not address how individualized CAPs and their temporal profiles can be assessed in relation to behavior, focusing on an identical number of clusters at both levels. In contrast, we focused on identifying reproducible spatial patterns of neural co-activation (CAPs) at the group level and quantifying a reproducible feature set of temporal profiles of the CAPs at the single subject level, therefore providing a unified framework to evaluate individual differences using a joint analysis of state and trait variations of neural co-activation. Similarly, several brain states were estimated at the group level using another dynamic functional connectivity approach in [24], whereas they could identify the time-points when the state was active, allowing the estimation of FO for each state and for each subject.

Collectively, these results highlight that state-trait CAP dynamics are reproducible at the single subject level across permutations and splits (**Fig. 3, S9 Fig**). For context, the statistics reported here (**Fig. 3C**) demonstrate higher reproducibility than the meta-analytic estimate for group-level reproducibility of area-to-area functional connectivity matrices [51]. Still, the observed ICC values are fairly low, reflecting a notable variance between days. This indicates that while trait-like features are most dominant factors loaded on the general motif of individual variance, the observed state variance (between days) also contributes to this general motif. In this context, the fairly low range of ICC reflects the notable amount of within-subject variance at the time-scale of days, and supports our framework of state-trait components that together identify neural phenotypes. Similarly, Yang et al. found that CAPs identified at individual level were unstable over time across the ten scans (~ 30 minutes/scan) except a few subjects, and subject-specific CAPs became more reliable and individual specific when integrating data with longer duration [55]. Therefore, we argue that such temporal fluctuations within subjects can bring critical insights into individual-specific brain organizations. Reducing the number of neural features into a reproducible set of CAPs may enable a more robust and reproducible mapping between neural features and behavior. In other words, we hypothesize that further optimization of reproducible data-reduced neural features presents a critical step toward mapping rs-fMRI signals to healthy and clinically-relevant behavioral variation and obtaining robust neuro-behavioral models."

Comment 5. Overall, the figures contain too much information. There are many parts that are unnecessary in delivering the main findings. I suggest removing many figures that assess the validity of the models or which purely describe observations that should be observed by the nature of the model. For example, Fig. 5 could be moved to supplement. The three distributions "should" be different across subgroups because the clustering algorithm found clusters that accentuate differences. Compared to the information it conveys, it's a too-busy figure. Fig. 1B-E could also be removed/moved to supplement because the importance of these figures is much less than Fig. 1G or 1H.

Response: As suggested by the reviewer, we moved Fig. 5 to S16 Fig. We moved Fig. 1C-E to S3 Fig and revised Figure 1 as follows.

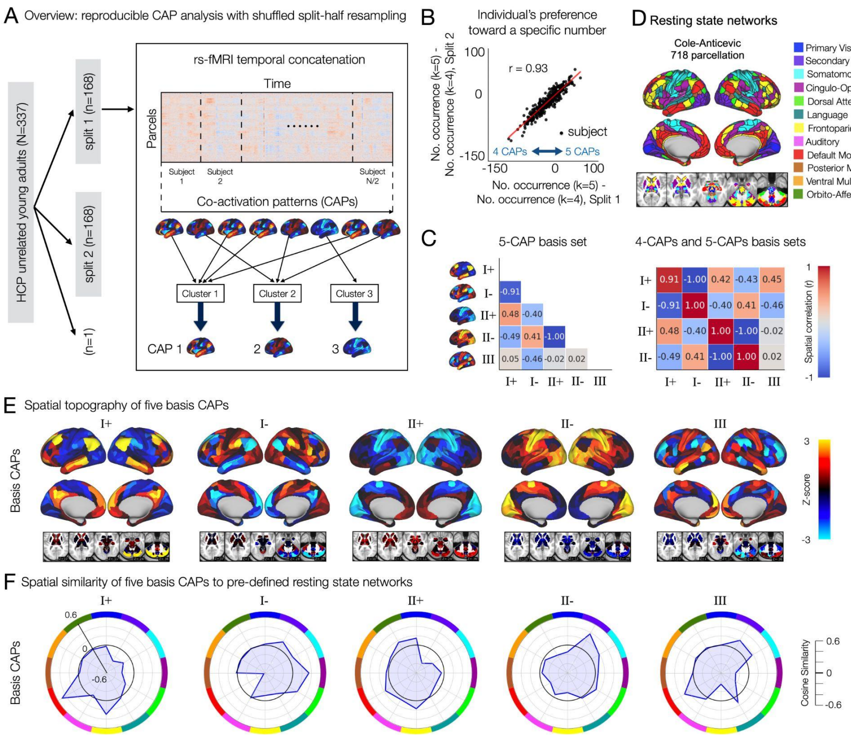

Figure 1. A reproducible set of co-activation patterns (CAPs) in the whole-brain rs-fMRI involve recurring mixed representations of canonical resting state networks. (A) Analysis overview. In each permutation, 337 subjects are randomly split into two equal-sized groups. Within each split, a parcel-by-time array of rs-fMRI data is temporally concatenated across subjects. Time-frames are clustered based on spatial similarity using K-means clustering. The number of clusters (k) is estimated for each split. Each CAP is obtained as the centroid of each cluster (Supplementary Fig.S1). (B) Individual's statistical preference toward a specific number of CAPs (k) is reproducible. In each split, an individual's preference toward a specific number was quantified using the number of permutations that resulted in a specific solution (eg. 4 CAPs or 5 CAPs) across 1,000 permutations. Specifically, we compute the difference (occurrence of k=5) - (occurrence of k=4) for each subject (Methods). (C) Spatial correlation of the 5-CAP basis set (left) and between the 4-CAP basis set and the 5-CAP basis set (right). r values were rounded to the nearest 2 decimal digits. (D) Cole-Anticevic Brain Network Parcellation (CAP-NP) (43). (E) Spatial topography of 5 basis CAPs. (F) Spatial similarity of the 5 basis CAPs to canonical resting state networks, pre-defined using the CAB-NP parcellation in (D).

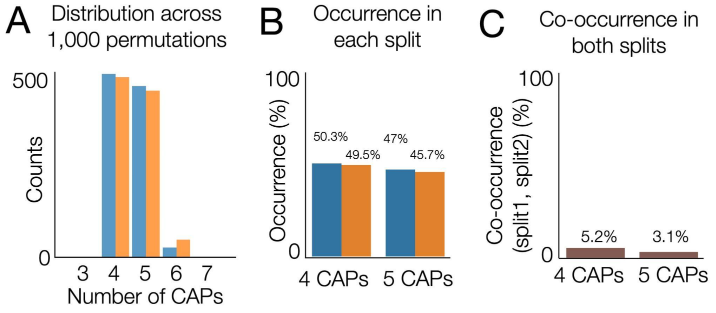

S3 Fig. Occurrence of CAPs across permutations. (A) The estimated number of CAPs (k) in each split across 1,000 permutations. (B) Occurrence rate (%) of k=4 or k=5 solutions in each split. (C) Co-occurrence rate (%) of k=4 or k=5 solutions in both splits.

Comment 6. I consider Fig. 7A-C misleading. The probability of CAP 3 occurrence "should" be associated with neural PCs because neural PCs were derived from CAP features which include the FO of CAP 3. Thus, significant relationships between CAP 3 occurrence and neural PCs are expected. However, with these figures visualized as such in the main figure, it may give a misleading impression that this is an interesting finding rather than the description of the neural PCs. On the other hand, Fig 7D is a finding. However, the sentence, "These results together indicate that the spatio-temporal properties of CAP 3 contribute to the positive correlation between the neural PC 1 and the behavioral PC 1 (Fig. 6G).", is not warranted because the contribution of CAP 3 FO in neural PC1 - behavioral PC 1 relationship was not directly tested by the statistical model.

Response: To better clarify and correct the flaw, we revised Fig. 7 and the texts as follows.

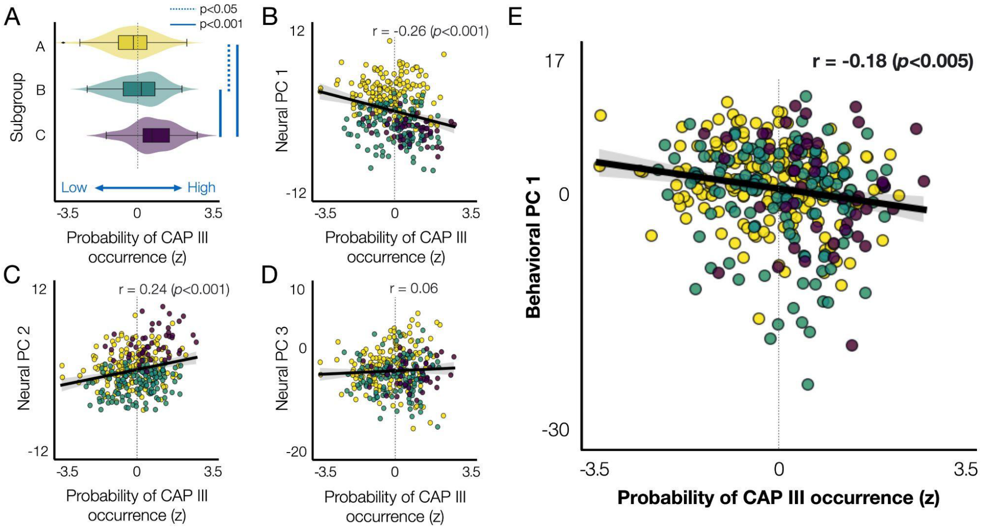

Figure 7. The probability of CAP III occurrence is associated with the neural and behavioral PCs. (A) The probability of CAP III occurrence (x-axis) for each individual, which can be interpreted as an individual's preference to have CAP III, was evaluated by the difference in the occurrence of 4 CAPs versus 5 CAPs, as described in Fig. 1B. For each subject, we computed the number of permutations (occurrence out of 1,000 permutations) when 4 CAPs were estimated and the number of permutations for the same subject to be involved when 5 CAPs were estimated. Then, for each subject, we compared the difference in the occurrence (**Δ** Occurrence = Occurrence(k=5) - Occurrence(k=4)) from each split. Then, for each individual, the **Δ** Occurrence was averaged over two splits. Finally, the within-subject average **Δ** Occurrence was normalized across subjects to z-scores. Individuals were color-coded by subgroups defined using the hierarchical clustering of 30 neural features (Fig. 4). (B)-(D) Scatter plots of individuals' preference to have CAP III with respect to the individual scores on the neural PC 1 (B), neural PC 2 (C), neural PC 3 (D), and behavioral PC 1 (E).

(p. 13) Results. "There was no relationship to individual scores of neural PC 3 (Fig. 7D). There was a weak negative correlation between the probability of CAP III occurrence and individual scores of behavioral PC 1 (r = -0.18, p<.005; Fig. 7E). We found no correlation between the probability of CAP III occurrence and behavioral PCs 2 and 3. These results together indicate the association of spatio-temporal properties of CAP III with the neural PCs and the behavioral PC 1."

Comment 7. In Fig. 2, shouldn't Fig 2A have 4 rows (ECi, with i = 1 to 4) and 5 columns instead of 5 x 5? Also, why is it 502 permutations?

Response: Thank you for finding the error. We corrected Fig 2A to have 4 rows and 5 columns. In this analysis, among 1,000 permutations, we only take permutations that resulted in 4-CAP solutions using the elbow method, which was 502 permutations for this data. The remaining 498 permutations mostly resulted in 5-CAP solutions, and rarely 6- or 7-CAP solutions. We clarified this in Fig. 2 Caption.

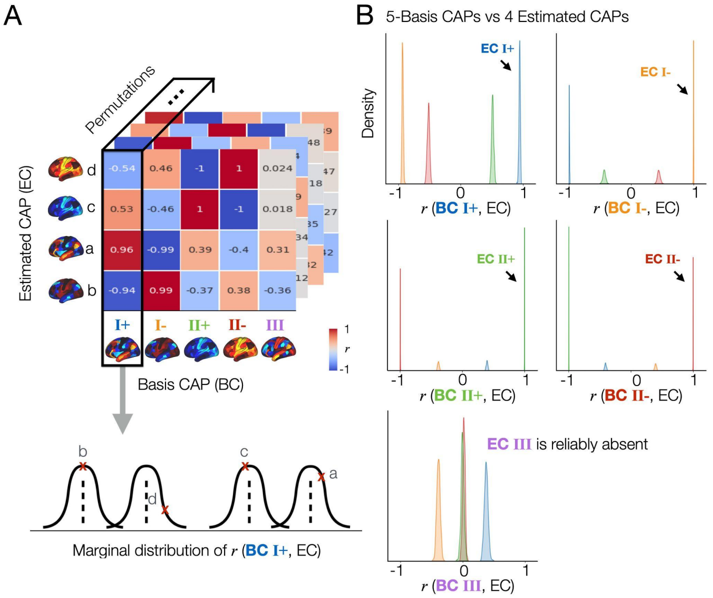

Figure 2. "**The spatial patterns of the CAPs estimated across split-half permutations are reproducible, demonstrating the consistent absence of a specific spatial pattern (CAP III) in one split but not in another split across permutations.** (A) Proof of concept. In this figure, we demonstrate "whenever 4 CAPs are estimated from a split data, are their spatial patterns reproducible across the permutations", and "if there is a specific CAP state that is reproducibly missing in 4-CAP solutions when compared to the 5-CAP solutions". To address these, first, Among 1,000 permutations, we only take permutations that resulted in 4-CAP solutions using the elbow method, which was 502 permutations in this data. The remaining 498 permutations mostly resulted in 5-CAP solutions, and rarely 6- or 7-CAP solutions as shown in (**S3 Fig**). Spatial similarity (r, correlation coefficient) is computed between each of the estimated CAPs (EC; denoted as a, b, c, and d) and a given basis CAP (BC)."

Comment 8. The authors succinctly described their analyses in the methods and used Figs. S1 and S2 to detail their workflow. However, it did not contain much of a "narrative". I had a hard time understanding why such a method was selected, why such a step was important, how such a method was used to test what, etc. I think the manuscript would improve in terms of readability if the methods were explained rather than simply described.

Response: We absolutely agree. We added a detailed description of the methods in the main text as follows.

(p.20-23) **Materials and Methods.**

Human Connectome Project (HCP) dataset [45]. Participants were recruited from Washington University (St. Louis, MO) and the surrounding area. We selected participants from the S1200 release of the HCP who had no family relations, resulting in a total of 337 participants included in our analyses. The dataset contains resting-

state fMRI data from 180 females and 157 males, with age range 22-37 (mean age=28.6, SD=3.7), 90% right-handed. Whole-brain echo-planar imaging data were collected with a 32 channel head coil on a modified 3T Siemens Skyra (Connectome Skyra) at WashU with time to repetition (TR)=720 ms, time to echo (TE)=33.1 ms, flip angle=52, bandwidth=2,290 Hz/pixel, in-plane field of view (FOV)=208 × 180 mm, 72 slices, and 2.0 mm isotropic voxels, with a multi-band acceleration factor of 8. rs-fMRI blood-oxygen-level-dependent (BOLD) images were collected over 2 days. On each day, 2 runs (14.5 min/run) of rs-fMRI were collected with opposite phase encoding directions (L/R and R/L). Subjects were instructed to keep eyes open with fixation on a cross-hair. Task-based imaging data were also collected, but not used in the present study. Structural MRIs were collected using the following parameters: T1-weighted (0.7 mm isotropic resolution, TR=2,400 ms, TE=2.14 ms, flip angle=8, in-plane field of view=224 × 224) and T2-weighted (0.7 mm isotropic resolution, TR=3,200 ms, TE=565 ms, variable flip angle, in-plane field of view=224 × 224). Find additional details about the dataset in [63].

Data preprocessing. We preprocessed rs-fMRI using the following steps corresponding to the steps advanced by the HCP consortium: i) the 'minimal preprocessing' pipeline outlined by [64], which involves intensity normalization, phase-encoding direction unwarping, motion correction, and spatial normalization to a standard template MSMAll [65], Angular Deviation Penalty (ADP) version; ii) High-pass filtering (0.009 Hz); iii) ICA-FIX for artifact removal [66]. Next, the 'minimally preprocessed' rs-fMRI in each run was represented in the Connectivity Informatics Technology Initiative (CIFTI) file format that combines surface-based data representation for cortex and volume-based data for subcortex gray matter locations (i.e. 'grayordinates'). Additional analyses were performed with Workbench v1.2.3 and Matlab 2014b (The Mathworks), using Quantitative Neuroimaging Environment & Toolbox (QuNex) [23,47].

Previous studies focusing on CAP analysis showed consistent CAP properties across the voxel and region levels [37,67]. To analyze CAPs at a low dimension space and to reduce the computational burden of CAP analysis that treats every 3-dimensional time-frame in the clustering process, we applied the Cole-Anticevic Brain Network Parcellation (CAB-NP) parcellation [46]. The CAP-NP parcellation is comprised of (i) 180 bilateral cortical parcels (a total of 360 across both left and right hemispheres), consistent with the Human Connectome Project's Multi-Modal Parcellation (MMP1.0) [65], and (ii) 358 subcortical parcels defined using resting-state functional BOLD covariation with the cortical network solution [46]. To remove any potential artifact at the onset/offset of each run, the first 100 frames were removed from every rs-fMRI run for each subject. To normalize rs-fMRI data in each run, the mean of each run was removed from each time series. Subsequently, rs-fMRI BOLD runs were concatenated in order of acquisition (rs-fMRI runs 2-1-4-3, R/L first, then L/R), resulting in a 4,000 × 718 array of rs-fMRI data for each subject.

CAP analysis. We identified moment-to-moment changes in the whole brain rs-fMRI BOLD signals at each time point and quantified the spatial patterns of co-activation (CAPs) across individuals, as well as individual variations in CAP temporal organization [33]. The analytic framework proposed in this study is described in **S1 Fig** and implemented using Python 3.6.15 using the Yale High Performance Computing resources. In each permutation, N=337 subjects are randomly split into two equal-sized groups (n=168, non-overlapping subjects). We used the shuffled split-half resampling strategy for several reasons. First, applying K-means clustering once to the concatenated time-series of the entire sample results in single values of neural measures per subject. Using such a simple approach, the individual reliability of CAP measures from the concatenated time-series across various sample populations could not be quantified. Our approach allowed for the estimation of statistical reproducibility of neural measures for each subject, when a subject was included in different sample

populations, therefore reducing potential sampling bias. In addition, we could use the same split-half resampling scheme for the cross-validation of neuro-behavioral association analysis, by training a neuro-behavioral model from split 1 data and testing the model on the remaining split 2 data.

Within each split, a 4,000 × 718 array of preprocessed rs-fMRI data are temporally concatenated across subjects. The following steps were performed for each split data using *scikit-learn 1.3.2* with Python. (i) Time-frames with excessive motions (Frame displacement >0.5 mm) are scrubbed [23,68,69]. The HCP minimal preprocessing pipeline included motion correction [64]; therefore, we avoided using a too conservative threshold for motion scrubbing and retained as many potentially useful frames as possible in our analysis. (ii) The remaining time-frames are clustered based on spatial similarity using the K-means clustering algorithm with Lloyd's algorithm, varying the number of clusters (k) from 2 to 15. (iii) An optimal number of clusters $\hat{k}$ is estimated. (iv) Using the K-means solutions with the optimal number $\hat{k}$, CAPs are defined as the cluster centroids, by parcel-wise averaging of the time-frames within each cluster.

Number of clusters. While the estimation of the number of clusters k is critical in CAP analysis, the field is lacking a consensus on the optimal criterion to determine it [70]. Earlier works used predefined arbitrary numbers ranging from 6 to 30 and reported that CAPs estimated using a small k are consistently found in results using a larger k [7,37,71]). It has been suggested that a large k may reduce cluster stability, for example, when a small number of time-frames are allocated to a cluster due to a short rs-fMRI acquisition duration [72]. Yang et al. calculated the silhouette scores for the clustering results varying k from 2 to 21 in both group and individual level analyses and chose k=4 as a trade-off to ensure the reliable estimation of spatiotemporal dynamics [55].

To find an optimal number, we used an approach that considers a trade-off between the number of clusters and within-cluster similarity (e.g. silhouette criteria [73]). We observed that the silhouette score was monotonically decreasing with the increase of k (**S2 Fig**), in agreement with Yang et al. [37]. In our study, to determine an optimal number of clusters, we varied the number of clusters (k) from 2 to 15. For each k, the K-means clustering was initialized using the k-means++ algorithm, by selecting randomly-generated centroids using sampling based on an empirical probability distribution of the points' contribution to the overall inertia. Inertia was defined as the sum of squared distances of samples to their closest cluster center. The maximum iteration for a single run was set to 1,000 to avoid that the algorithm stops before fully converging. Silhouette coefficient is estimated for the K-means solution using each k. Finally, an optimal number $\hat{k}$ is determined by applying the elbow method for the estimated Silhouette scores. The elbow point was defined when we observe a significant change in the rate of decrease of the Silhouette score as k increases, using the KneeLocator class in Python's kneed package.

Basis CAP generation. The occurrence rate (%) of the $\hat{k}$ =a solution was calculated by the number of permutations resulting in *a* clusters divided by the total number of permutations (1,000). Co-occurrence rate (%) of the $\hat{k}$ =a solution in both splits was determined by the number of permutations resulting in the same number of clusters divided by the total number of permutations. See Part (1) of the procedure diagram in **S4 Fig**).

A set of basis CAPs can be obtained using the agglomerative hierarchical clustering of the CAPs estimated from the permutations resulting in the same number of clusters ($\hat{k}$ =a), as follows. For each split, let's first denote that h_{MAX}/1,000 permutations resulted in $\hat{k}$ =a solution. (1) We collect the $\hat{k}$ CAPs (P × $\hat{k}$) and concatenate them across h_{MAX} permutations to produce a (P × $\hat{k}$ h_{MAX}) array, where P is the number of parcels. (2)

Agglomerative hierarchical clustering is applied to this array to identify $\hat{k}$ clusters based on spatial similarity. (3) In each cluster, co-activation values in each parcel are averaged across CAPs assigned to the same cluster, generating an average (basis) CAP. (4) The values in each parcel of the basis CAP are normalized to Z-scores, using the mean and standard deviation across the parcels in the whole brain. Steps (3) and (4) are repeated for all $\hat{k}$ clusters, resulting in $\hat{k}$ basis CAPs. See Part (2) of the diagram in **S2 Fig**.

Individual preference for a specific CAP. The probability of CAP occurrence, which can be interpreted as an individual's probabilistic preference for a specific CAP, was quantified by examining the number of permutations that resulted in a specific solution k. To do this, we compared the probability to have k CAPs involving the CAP of interest and the probability to have k-1 CAPs without involving the CAP of interest, assuming a reproducible estimation of spatial topography of k CAPs across permutations, similar to the approaches comparing full and reduced models. Specifically, across 1,000 split-half permutations, a subject is involved in split 1 data for p_1 permutations and in split 2 data for $p_2 = 1000 - p_1$. Then, when only considering split 1 data from these p_1 permutations, we can compute the number of permutations that resulted in k and the number of permutations that resulted in k-1. In each split, for each subject, we compute the difference (occurrence of k CAPs) minus (occurrence of k-1 CAPs) to quantify an individual's preference for a specific CAP. To associate these with behavioral variables (normalized), we normalized the individual's probabilistic preference for a specific CAP using Z-transformation across subjects.

Neural dimension reduction. To identify the principal geometry of the state-trait neural feature space, thirty neural features are estimated for each individual: three neural measures (FO, mean DT, and var DT) × five CAPs (I+, I-, II+, II-, and III) × 2 days. These neural features were collected across subjects to create a subject-by-feature matrix. Two analyses are performed on this subject-by-feature matrix. First, agglomerative hierarchical clustering was applied to the feature matrix, using *scikit-learn 1.3.2*. The ward linkage criterion with Euclidean metric was used to minimize the variance of the clusters being merged. The number of clusters was determined using a distance cut-off value of 70% of the final merge in the dendrogram. Second, PCA was applied to this subject-by-feature matrix to estimate the principal geometry of this state-trait feature space identifying subgroups.

> **Comment 9.** When estimating CAPs, the authors used a shuffled split-half resampling strategy across multiple permutations. However, what was the motivation for taking this approach, rather than, for example, applying K-means clustering just once to the concatenated time series of the entire sample? For the permutation, is the reason because K-means clustering estimation gave different outputs every time it was run? Is split-half analysis due to test for reliability? Please justify in the manuscript.

Response: Thank you for the comment. We justified this in the revised manuscript as follows.

(p. 21) Methods. "CAP analysis. We identified moment-to-moment changes in the whole brain rs-fMRI BOLD signals at each time point and quantified the spatial patterns of co-activation (CAPs) across individuals, as well as individual variations in CAP temporal organization [33]. The analytic framework proposed in this study is described in **S1 Fig** and implemented using Python 3.6.15 using the Yale High Performance Computing resources. In each permutation, N=337 subjects are randomly split into two equal-sized groups (n=168, non-overlapping subjects). We used the shuffled split-half resampling strategy for several reasons. First, applying K-means clustering once to the concatenated time-series of the entire sample results in single values of neural

measures per subject. Using such a simple approach, the individual reliability of CAP measures from the concatenated time-series across various sample populations could not be quantified. Our approach allowed for the estimation of statistical reproducibility of neural measures for each subject, when a subject was included in different sample populations, therefore reducing potential sampling bias. In addition, we could use the same split-half resampling scheme for the cross-validation of neuro-behavioral association analysis, by training a neuro-behavioral model from split 1 data and testing the model on the remaining split 2 data.**"**

Comment 10. In Methods, "Lastly, an k-CAP basis set was obtained by using the agglomerative hierarchical clustering of the CAPs estimated from all permutations (Supplementary Fig. S2)." Following this sentence, please describe the steps conceptually. This is an important method but Fig. S2 is insufficient to deliver intuitively what has been done.

Response: We added a detailed description in a separate subsection in **(p.22) Methods. Basis CAP generation.** Please refer to our response to your **Comment 8** for the revised texts in the Methods section.

Comment 11. Explain the elbow method that was used to determine the optimal K. What criteria determined the "elbow"?

Response: We added a detailed description in a separate subsection in **(p.22) Methods. Number of Clusters.** Please refer to our response to your **Comment 8** for the revised texts in the Methods section. In addition, we performed 10 additional permutations for the split-half CAP analysis and quantified the silhouette scores for each split varying k from 2 to 15 to show. The result is now included as a **S2 Fig** in the revised manuscript.

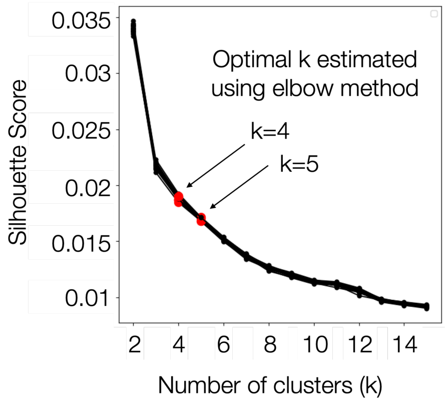

S2 Fig. Quality of the K means clustering solution in CAP analysis. Silhouette scores were estimated across different numbers of clusters (k) from the K-means clustering solution from a split data. Results from 10 permutations (two split-halves in each permutation) are shown. Optimal k values were estimated using the elbow method for the Silhouette scores and are highlighted in red.

(p. 4) Results. **"**The time-frames are clustered based on spatial similarity using K-means clustering, where the number of clusters (k) is estimated for each split using the elbow method varying k from 2 to 15 (see the estimated Silhouette scores from the K-means clustering solutions in (**S2 Fig**). **"**

Comment 13. Figs 2 and 3 highlighted the reliability of CAP states and their features. Additionally, I was curious about the FO, mean DT, and var DT distributions of three CAP states. For example, were the fractional occupancies of CAPs hugely varied across participants? Were the fractional occupancies of CAPs 1 and 2 much higher than the fractional occupancy of CAP 3 in general?

Response: In the **S13 Fig** (formerly Figure 5), we show the distributions of FO, mean DT and var DT values across individuals and across two days. We hope that this figure could answer these and other related questions. For example, the FO of CAPs I and II showed higher mean and variability than the FO of CAP III. We observed the same patterns in the mean DT and var DT. We explicitly mentioned this finding in the text, as follows.

(p. 10) Results. "The assessment of individual distributions of each neural measure supported these findings (**S13 Fig**). In addition, we found that the FO of CAPs I and II have overall a higher mean and variability than the FO of CAP III. We observed the same patterns in the mean DT and var DT (**S13 Fig**)."

Comment 14. (a) Pg 5. Left column "The correlation of individual neural measures between day 1 and day 2 was ...": Are these r values of between-days reliability significant, when compared to a null distribution (non-parametric permutation test)? **(b)** Also, how are these values (mean r = 0.41, 0.41, and 0.38 for FO, mean DT, and var DT) different from the values reported in Fig. 3D (r = 0.5, 0.45, and 0.41)?

Response to (a): These r-values were measured by Pearson's correlation coefficients, assuming that the r-values are normally distributed and that there is a linear relationship between them. The values were considered significant when the corresponding two-sided uncorrected p-values are less than 0.001. We clarified this in the Figure 3 caption, as follows.

Response to (b): In Fig 3D, the three scatter plots of individual FO, mean DT and var DT show the results specific to CAP I+, as written on the left top corner of each plot and in the caption. In the text (page 5 of the reviewed manuscript version 1), the reported values are the mean and standard deviation values calculated across the results from all five CAPs, as written in the text "(mean +/- SD over five CAPs)". To avoid any further confusion, we updated the figure caption and the text, as follows.

Caption. Figure 3. (D) (D) Test-retest reliability of neural measures between two days of scan. (top) For CAP I+ state, we show scatter plots of individual FO, within-subject mean and variance of DT between days 1 and 2. Linear fitting line (red) is shown for each scatter plot. r-value is measured by Pearson's correlation coefficient and considered significant when the corresponding two-sided p-value is less than 0.001. (bottom) For the remaining four CAP states, the same scatter plot analysis was repeated (**S10 Fig**). We summarize the estimated r-values from all CAPs in the bar plot.

(p.7) Results. "First, we measured the test-retest reliability of the neural measures using a linear regression (**Fig. 3D**). For each CAP, we found a moderate low correlation ($r \leq 0.5$) of individual neural measures between day 1 and day 2 (**Fig. 3D**). CAP I+ showed the highest between-day reliability and CAP III was the lowest. See **S10 Fig** for the scatter plots from the other four CAP states. When calculating the mean and SD of correlation across all CAPs, the between-day correlation is 0.41 ± 0.07 for FO, 0.41 ± 0.06 for mean DT, and 0.38 ± 0.07 for var DT."

Comment 15. Fig. 3B: The figure well illustrates across-permutation reliability and across-split reliability. However, if I'm understanding correctly, if between-day reliability is high - as roughly r = 0.4 - then shouldn't the Day 2 color scale roughly show a green-red-blue gradient from top to bottom? (or is it already showing but I'm just not seeing the gradation?)

Response: We think that this observed visual effect may be attributed, at least in part, to the utilization of a saturated color gradation in Figure 3 to optimize visual clarity across rows (subjects). In Supplementary Figure S6, we present the same results using an unsaturated color gradation. Given that employing the colormap for the entire range of data failed to provide distinct contrast across subjects, we opted for the saturated colormap in the main text. This decision has been elucidated in the revised figure caption as follows.

Caption. Figure 3. (B) Stability of individual mean DT of CAP I+ across permutations and across two days. Individual subjects were rank-ordered from top to bottom using the split 1 data from Day 1. While the estimated mean DT values spanned from 0 to 6, the dataset exhibited sparse occurrences in the distribution tails. To enhance visual clarity across rows (subjects), a saturated colormap was employed. For an alternative representation of the same data using an unsaturated colormap, refer to **S9 Fig**. We also found that individual Var DT and individual FO for these CAPs are reproducible across permutations and two days (**S9 Fig**).

Comment 16. Pg 3. Last sentence of the left column: change "cosign similarity" to "cosine similarity".

Response: We corrected the typo.

Comment 17. For simplicity, I don't find the need to additionally define "time-consecutive segment (c)". Dwell times can be explained without the "additional" explanation of the time-consecutive segment.

Response: We removed the definition of “time-consecutive segment (c)”.

Response to Reviewer #3 (Golia Shafiei & Ted Satterthwaite)

Comment 1. Methods detail. We were surprised that the methods were fairly sparse. While many of the methods were introduced in the results, they received relatively dedicated treatment in the methods section. This section could be expanded (either in the main text or the supplement) to allow readers to understand the approach in greater detail. For example, there is limited detail regarding the clustering algorithms and the associated parameters. (E.g., what was the distance metric used for agglomerative clustering?).

Response: We absolutely agree with this and added a detailed description of the methods as follows.

(p.20-23) Materials and Methods.

Human Connectome Project (HCP) dataset [45]. Participants were recruited from Washington University (St. Louis, MO) and the surrounding area. We selected participants from the S1200 release of the HCP who had no family relations, resulting in a total of 337 participants included in our analyses. The dataset contains resting-state fMRI data from 180 females and 157 males, with age range 22-37 (mean age=28.6, SD=3.7), 90% right-handed. Whole-brain echo-planar imaging data were collected with a 32 channel head coil on a modified 3T Siemens Skyra (Connectome Skyra) at WashU with time to repetition (TR)=720 ms, time to echo (TE)=33.1 ms, flip angle=52, bandwidth=2,290 Hz/pixel, in-plane field of view (FOV)=208 × 180 mm, 72 slices, and 2.0 mm isotropic voxels, with a multi-band acceleration factor of 8. rs-fMRI blood-oxygen-level-dependent (BOLD) images were collected over 2 days. On each day, 2 runs (14.5 min/run) of rs-fMRI were collected with opposite phase encoding directions (L/R and R/L). Subjects were instructed to keep eyes open with fixation on a cross-hair. Task-based imaging data were also collected, but not used in the present study. Structural MRIs were collected using the following parameters: T1-weighted (0.7 mm isotropic resolution, TR=2,400 ms, TE=2.14 ms, flip angle=8, in-plane field of view=224 × 224) and T2-weighted (0.7 mm isotropic resolution, TR=3,200 ms, TE=565 ms, variable flip angle, in-plane field of view=224 × 224). Find additional details about the dataset in [63].

Data preprocessing. We preprocessed rs-fMRI using the following steps corresponding to the steps advanced by the HCP consortium: i) the 'minimal preprocessing' pipeline outlined by [64], which involves intensity normalization, phase-encoding direction unwarping, motion correction, and spatial normalization to a standard template MSMAll [65], Angular Deviation Penalty (ADP) version; ii) High-pass filtering (0.009 Hz); iii) ICA-FIX for artifact removal [66]. Next, the 'minimally preprocessed' rs-fMRI in each run was represented in the Connectivity Informatics Technology Initiative (CIFTI) file format that combines surface-based data representation for cortex and volume-based data for subcortex gray matter locations (i.e. 'grayordinates'). Additional analyses were performed with Workbench v1.2.3 and Matlab 2014b (The Mathworks), using Quantitative Neuroimaging Environment & Toolbox (QuNex) [23,47].

Previous studies focusing on CAP analysis showed consistent CAP properties across the voxel and region levels [37,67]. To analyze CAPs at a low dimension space and to reduce the computational burden of CAP analysis that treats every 3-dimensional time-frame in the clustering process, we applied the Cole-Anticevic Brain Network Parcellation (CAB-NP) parcellation [46]. The CAP-NP parcellation is comprised of (i) 180 bilateral cortical parcels (a total of 360 across both left and right hemispheres), consistent with the Human Connectome Project's Multi-Modal Parcellation (MMP1.0) [65], and (ii) 358 subcortical parcels defined using resting-state functional BOLD covariation with the cortical network solution [46]. To remove any potential artifact at the onset/offset of each run, the first 100 frames were removed from every rs-fMRI run for each subject. To normalize rs-fMRI data in each run, the mean of each run was removed from each time series. Subsequently, rs-fMRI BOLD runs were concatenated in order of acquisition (rs-fMRI runs 2-1-4-3, R/L first, then L/R), resulting in a 4,000 × 718 array of rs-fMRI data for each subject.

CAP analysis. We identified moment-to-moment changes in the whole brain rs-fMRI BOLD signals at each time point and quantified the spatial patterns of co-activation (CAPs) across individuals, as well as individual variations in CAP temporal organization [33]. The analytic framework proposed in this study is described in **S1 Fig** and implemented using Python 3.6.15 using the Yale High Performance Computing resources. In each permutation, N=337 subjects are randomly split into two equal-sized groups (n=168, non-overlapping subjects). We used the shuffled split-half resampling strategy for several reasons. First, applying K-means clustering once to the concatenated time-series of the entire sample results in single values of neural measures per subject. Using such a simple approach, the individual reliability of CAP measures from the concatenated time-series across various sample populations could not be quantified. Our approach allowed for the estimation of statistical reproducibility of neural measures for each subject, when a subject was included in different sample populations, therefore reducing potential sampling bias. In addition, we could use the same split-half resampling scheme for the cross-validation of neuro-behavioral association analysis, by training a neuro-behavioral model from split 1 data and testing the model on the remaining split 2 data.

Within each split, a 4,000 × 718 array of preprocessed rs-fMRI data are temporally concatenated across subjects. The following steps were performed for each split data using *scikit-learn 1.3.2* with Python. (i) Time-frames with excessive motions (Frame displacement >0.5 mm) are scrubbed [23,68,69]. The HCP minimal preprocessing pipeline included motion correction [64]; therefore, we avoided using a too conservative threshold for motion scrubbing and retained as many potentially useful frames as possible in our analysis. (ii) The remaining time-frames are clustered based on spatial similarity using the K-means clustering algorithm with Lloyd's algorithm, varying the number of clusters (k) from 2 to 15. (iii) An optimal number of clusters $\hat{k}$ is estimated. (iv) Using the K-means solutions with the optimal number $\hat{k}$, CAPs are defined as the cluster centroids, by parcel-wise averaging of the time-frames within each cluster.

Number of clusters. While the estimation of the number of clusters k is critical in CAP analysis, the field is lacking a consensus on the optimal criterion to determine it [70]. Earlier works used predefined arbitrary numbers ranging from 6 to 30 and reported that CAPs estimated using a small k are consistently found in results using a larger k [7,37,71]). It has been suggested that a large k may reduce cluster stability, for example, when a small number of time-frames are allocated to a cluster due to a short rs-fMRI acquisition duration [72]. Yang et al. calculated the silhouette scores for the clustering results varying k from 2 to 21 in both group and individual level analyses and chose k=4 as a trade-off to ensure the reliable estimation of spatiotemporal dynamics [55].

To find an optimal number, we used an approach that considers a trade-off between the number of clusters and within-cluster similarity (e.g. silhouette criteria [73]). We observed that the silhouette score was monotonically decreasing with the increase of k (**S2 Fig**), in agreement with Yang et al. [37]. In our study, to determine an optimal number of clusters, we varied the number of clusters (k) from 2 to 15. For each k, the K-means clustering was initialized using the k-means++ algorithm, by selecting randomly-generated centroids using sampling based on an empirical probability distribution of the points' contribution to the overall inertia. Inertia was defined as the sum of squared distances of samples to their closest cluster center. The maximum iteration for a single run was set to 1,000 to avoid that the algorithm stops before fully converging. Silhouette coefficient is estimated for the K-means solution using each k. Finally, an optimal number $\hat{k}$ is determined by applying the elbow method for the estimated Silhouette scores. The elbow point was defined when we observe a significant change in the rate of decrease of the Silhouette score as k increases, using the KneeLocator class in Python's kneed package.

Basis CAP generation. The occurrence rate (%) of the $\hat{k}$ =a solution was calculated by the number of permutations resulting in a clusters divided by the total number of permutations (1,000). Co-occurrence rate (%) of the $\hat{k}$ =a solution in both splits was determined by the number of permutations resulting in the same number of clusters divided by the total number of permutations. See Part (1) of the procedure diagram in **S4 Fig**).

A set of basis CAPs can be obtained using the agglomerative hierarchical clustering of the CAPs estimated from the permutations resulting in the same number of clusters ($\hat{k}$ =a), as follows. For each split, let's first denote that h_{MAX}/1,000 permutations resulted in $\hat{k}$ =a solution. (1) We collect the $\hat{k}$ CAPs (P × $\hat{k}$) and concatenate them across h_{MAX} permutations to produce a (P × $\hat{k}$ h_{MAX}) array, where P is the number of parcels. (2) Agglomerative hierarchical clustering is applied to this array to identify $\hat{k}$ clusters based on spatial similarity. (3) In each cluster, co-activation values in each parcel are averaged across CAPs assigned to the same cluster, generating an average (basis) CAP. (4) The values in each parcel of the basis CAP are normalized to Z-scores, using the mean and standard deviation across the parcels in the whole brain. Steps (3) and (4) are repeated for all $\hat{k}$ clusters, resulting in $\hat{k}$ basis CAPs. See Part (2) of the diagram in **S2 Fig**.

Individual preference for a specific CAP. The probability of CAP occurrence, which can be interpreted as an individual's probabilistic preference for a specific CAP, was quantified by examining the number of permutations that resulted in a specific solution k. To do this, we compared the probability to have k CAPs involving the CAP of interest and the probability to have k-1 CAPs without involving the CAP of interest, assuming a reproducible estimation of spatial topography of k CAPs across permutations, similar to the approaches comparing full and reduced models. Specifically, across 1,000 split-half permutations, a subject is involved in split 1 data for p_1 permutations and in split 2 data for $p_2 = 1000 - p_1$. Then, when only considering split 1 data from these p_1 permutations, we can compute the number of permutations that resulted in k and the number of permutations that resulted in k-1. In each split, for each subject, we compute the difference (occurrence of k CAPs) minus (occurrence of k-1 CAPs) to quantify an individual's preference for a specific CAP. To associate these with behavioral variables (normalized), we normalized the individual's probabilistic preference for a specific CAP using Z-transformation across subjects.

Neural dimension reduction. To identify the principal geometry of the state-trait neural feature space, thirty neural features are estimated for each individual: three neural measures (FO, mean DT, and var DT) × five CAPs (I+, I-, II+, II-, and III) × 2 days. These neural features were collected across subjects to create a subject-

by-feature matrix. Two analyses are performed on this subject-by-feature matrix. First, agglomerative hierarchical clustering was applied to the feature matrix, using *scikit-learn 1.3.2*. The ward linkage criterion with Euclidean metric was used to minimize the variance of the clusters being merged. The number of clusters was determined using a distance cut-off value of 70% of the final merge in the dendrogram. Second, PCA was applied to this subject-by-feature matrix to estimate the principal geometry of this state-trait feature space identifying subgroups.

Comment 2. Underlying biology. Although the analytical approach is a tour de force and is an important contribution to the field by itself, links to underlying biology received relatively scant attention. Contextualizing results in the context of systems/developmental neuroscience could further increase the impact of this study.

Response: To enrich the neuroscientific relevance and interpretation of our findings, we first extended our analysis to associate the first three neural PCs and the first three behavioral PCs. We ensured the reproducibility of our neuro-behavioral mapping using permutation and cross-validation. **Figure 6, S16 Fig** and **S17 Fig** were added as shown below. Then, we discussed these results in relation to underlying biology.

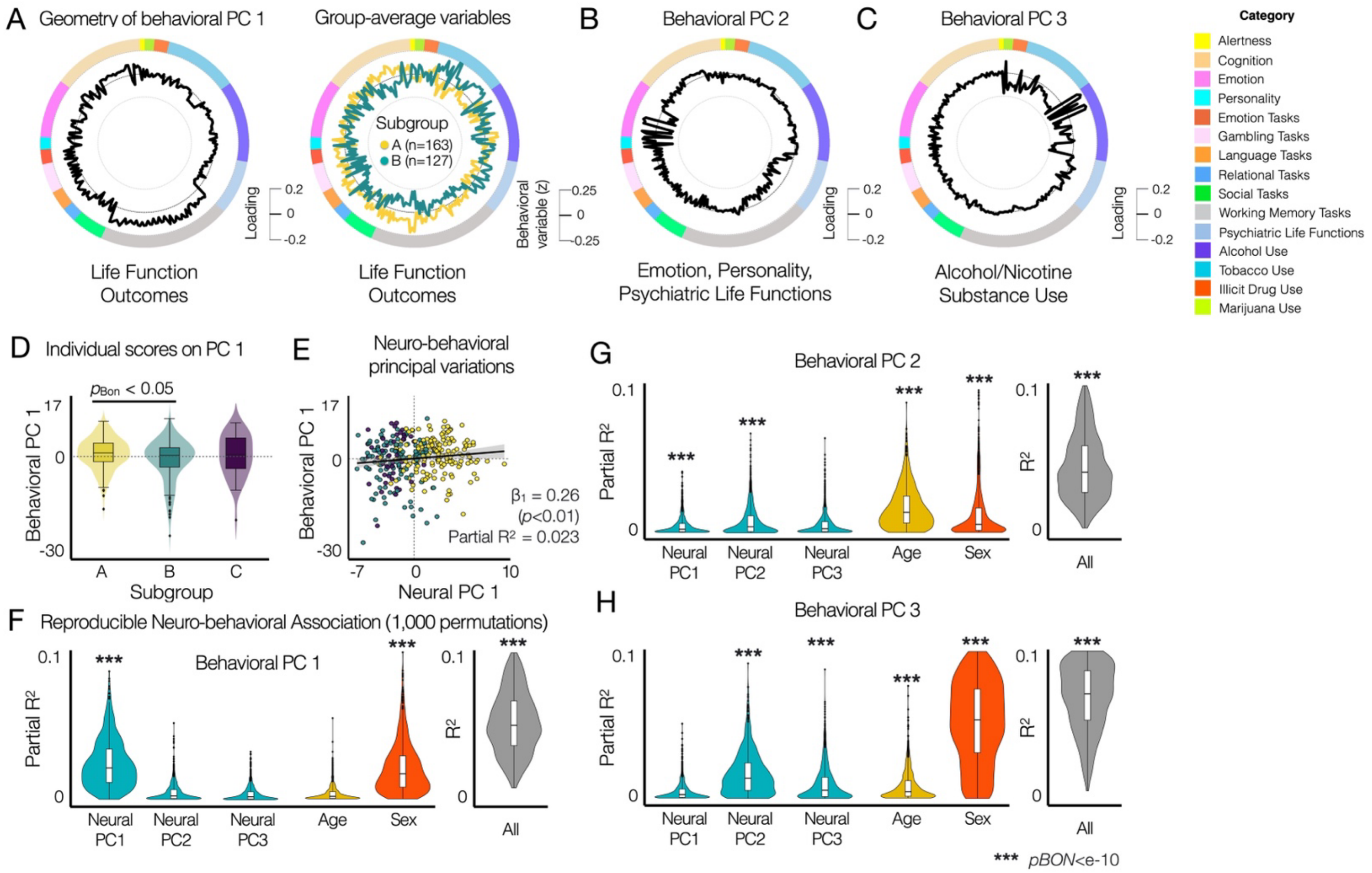

Figure 6. "**Principal variations of neural state-trait features co-vary with the principal variations of behavioral phenotypes, highlighting individual life function outcomes associated with emotion regulation, cognitive function and alcohol and substance use. (A)** The geometry of behavioral PC 1 (black, left circle) reflects the difference in group-average behavioral variables (standardized behavioral data, right circle) between

subgroups **A** (yellow) and **B** (green). Subgroup **C** is not shown because no significant group differences are found in (D). **(B)** The geometry of behavioral PC 2. **(C)** The geometry of behavioral PC 3. **(D)** Comparison of individual PC 1 scores between subgroups identified using neural state-trait measures (Fig. 4). Two-sample two-sided t-tests were performed between subgroups for each behavioral PC. p_{BON}: Bonferroni corrected p-values. **(E)** Multiple linear regression model of three neural PC 1 with two covariates (age and sex) showed that the neural PC 1 was associated with the behavioral PC 1 (Partial $R^2 = 0.023$, $\beta_1 = 0.26$, SE = 0.09, t = 2.8, p = 0.006), where multiple $R^2 = 0.041$, adjusted $R^2 = 0.026$, F(5, 331)=2.814, p-value = 0.017 for the full model. **(F-H)** Reproducibility analysis of the prediction of individual behavioral PC scores from neural PCs. In each permutation, PCA was performed for the neural and behavioral data from subjects in a random half of the entire sample (N=168). Parameters of multiple linear regression models with three neural PC 1 with two covariates (age and sex) were estimated to evaluate the predictability of each behavioral PC. p_{BON}: Bonferroni corrected p-values from F-tests."

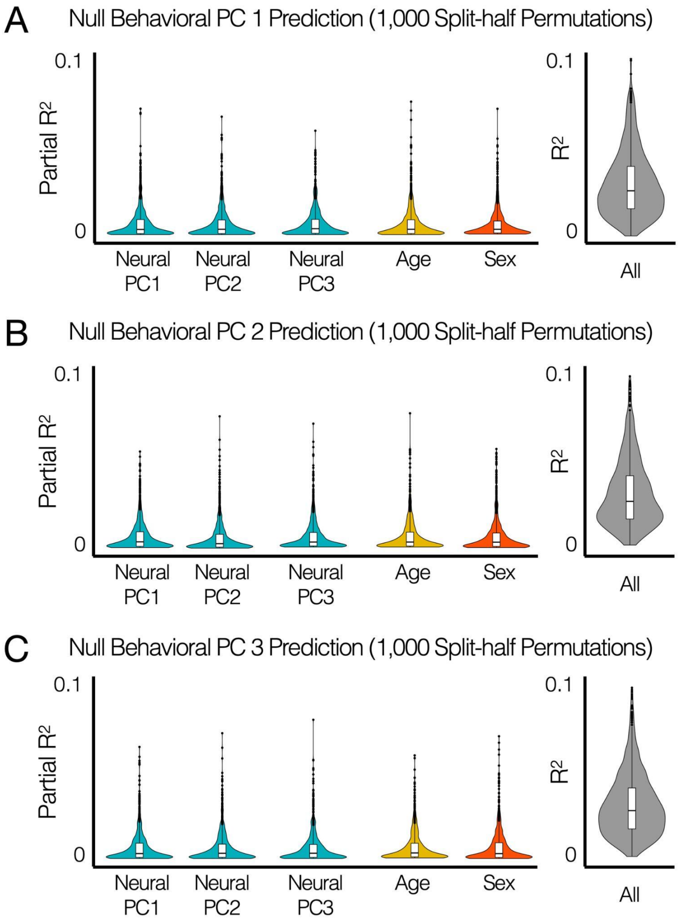

S15 Fig. Null data were generated by shuffling individual subjects in behavioral data. Null distributions of partial R^2 were estimated for each predictor in the neuro-behavioral association model trained from a split data across 1,000 split-half permutations."

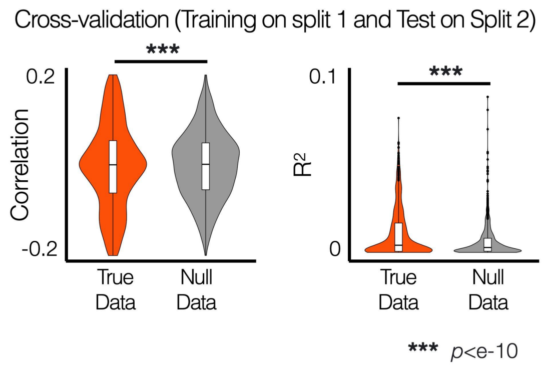

S16 Fig. Split-half permutation based cross-validation of the prediction model of predicting behavioral PC 1 from neural PCs. The multiple linear regression models were trained using split 1 data and tested on split 2 data in each permutation. Null data were generated by shuffling individual subjects in behavioral data."

(p. 13) Results. "**Reproducibility and Cross-validation of Low-Dimensional Neuro-Behavioral Association.**

To further evaluate the reproducibility of the neuro-behavioral association in our low-dimensional space found in **Fig. 6E**, we first performed the same multiple linear regression approach on a split data (random N=168) across 1,000 permutations. Null data were generated by shuffling individual subjects in each behavioral PC data (**S15 Fig**). For predicting behavioral PC 1, the resulting partial R^2 values were strongly reproducible across permutations (partial R^2 =0.025 ± 0.017 for neural PC1, p_{BON} <e-10, F-test; overall R^2=0.056 ± 0.024), as shown in **Fig. 6F**. Similar to the analysis using the entire data, sex was also a reproducible predictor of behavioral PC 1 (partial R^2 =0.02 ± 0.016, p_{BON} <e-10, F-test). Secondly, we used the multiple linear regression model trained from each split 1 data for predicting individual behavioral scores in the corresponding split 2 data across permutations (**S16 Fig**). While the overall prediction performance was relatively low, it was highly reproducible and significantly different from null data analysis (R^2=0.011 ± 0.013, p<e-10, F-test).

Next, we repeated the same analyses for predicting behavioral PCs 2 and 3. Neural PCs 1 and 2 showed reproducible association with behavioral PC 2 that highlights emotion, personality, and psychiatric life functions, whereas age and sex showed larger predictive power (**Fig. 6G**). For predicting behavioral PC 2, the estimated partial R^2 was 0.008 ± 0.01 for neural PC2, 0.018 ± 0.014 for age, and 0.013 ± 0.019 for sex (**Fig. 6G**). On the other hand, neural PC 2 was predictive of behavioral PC 3 highlighting a strong association with alcohol consumption habits and sex differences (**Fig. 6H**). For predicting behavioral PC 2, the estimated partial R^2 was 0.016 ± 0.014 for neural PC2 and 0.059 ± 0.036 for sex, whereas the overall R^2 for the full model was 0.098 ± 0.041 (**Fig. 6H**)."

(p. 19) Discussion. "Our results in the reproducible neuro-behavioral associations highlighted the neural relevance of behavioral PCs 1, 2 and 3. Individuals with higher cognition (behavioral PC 1) spent longer time at CAP I than at CAP II. The behavioral PC 2 showed an association with neural PCs 1 and 2, as well as age and sex. In relation to resting state functional connectivity, While CAPs I and II show differences in the regions of language, primary visual, and cingulo-opercular networks, the overall patterns of these CAP states involved opposing patterns of brain activity in the default mode and frontoparietal networks versus the regions belonging to the secondary visual, somatomotor and dorsal attention networks [61]. This suggests a relation to the cortical

hierarchy that spans from unimodal sensorimotor cortices to transmodal association cortices and a potential role in functional connectivity development during childhood through adolescence [62].”

Additional References:

[61] Larsen et al., A critical period plasticity framework for the sensorimotor-association axis of cortical development, Trends in Neuroscience, 2023

[62] Luo et al., Functional connectivity development along the sensorimotor-association axis enhances the cortical hierarchy, Nature Communications, 2024

Comment 3. Clustering quality. It is somewhat unclear what the quality of the clustering solution was. Did the authors consider quantifying this using a measure such as silhouette score? The same comment applies to the K-means solution for the CAP analysis.

Response: As described in the revised **Methods**, to determine the optimal number of clusters, we used an approach that considers a trade-off between the number of clusters and within-cluster similarity (e.g. silhouette criteria). Because we unfortunately did not save the data of estimated Silhouette scores across 1,000 permutations, we performed 10 additional permutations for the split-half CAP analysis and quantified the silhouette scores for each split varying k from 2 to 15. The result is now included as a **S2 Fig** in the revised manuscript.

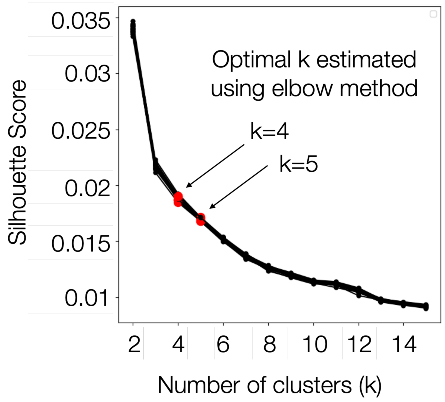

S2 Fig. Quality of the K means clustering solution in CAP analysis. Silhouette scores were estimated across different numbers of clusters (k) from the K-means clustering solution from a split data. Results from 10 permutations (two split-halves in each permutation) are shown. Optimal k values were estimated using the elbow method for the Silhouette scores and are highlighted in red.

(p. 4) Results. **“**The time-frames are clustered based on spatial similarity using K-means clustering, where the number of clusters (k) is estimated for each split using the elbow method varying k from 2 to 15 (see the estimated Silhouette scores from the K-means clustering solutions in (**S2 Fig**). **”**

Comment 4. Temporal censoring in preprocessing. The analysis workflow depicted in Fig S1 indicates that scrubbing was applied for the time frame selection procedure; this is not mentioned in the methods section. How did the scrubbing process influence the findings of CAPs analysis, especially analyses of state transitions &

dwell time, which would seem likely to be heavily impacted by the timeseries discontinuities introduced by scrubbing?

Comment 5. Individual differences in motion. Relatedly, did residual motion after scrubbing relate to the CAPS themselves, the subgroups, or the brain-behavior relationships? Including mean FD as a covariate would enhance confidence.

Response: Thank you for the comments, these are important points. To address these, we created a section "Impact of motion" and performed several additional analyses.

(p. 13) Results. "**Impact of motion.** To evaluate the impact of motion, the mean Frame Displacement (FD) was computed across time-frames for each subject. The estimated mean FD was 0.16 +/- 0.58 across subjects (N=337) . Note that we scrubbed time-frames with excessive motion (FD>0.5 mm) when estimating CAPs (**S1 Fig**). The average number of scrubbed time-frames across subjects was 76 +/- 192.6 (counts), which are 1.73 +/- 4.38 % of the total number of time-frames (4,400/subject before removing dummy scans) in each subject. More than 5% of total time-frames were scrubbed in 28/337 subjects (8.3%). We also measured the duration of motion (the number of consecutive time-frames with excessive motion) to assess whether there were long time-segments of motion, which might impact the estimation of temporal CAP profiles, especially analyses of CAP state transitions and dwell time. The length of motion-related continuous time-frames was 1.34 +/-0.38 time-frames on average across subjects. Repeating the analyses excluding these 28 subjects did not change the results, including the PCA of neural measures and the low-dimensional neuro-behavioral relationships (**S17 Fig**)."

n=309 subjects excluding 28 subjects with high motion

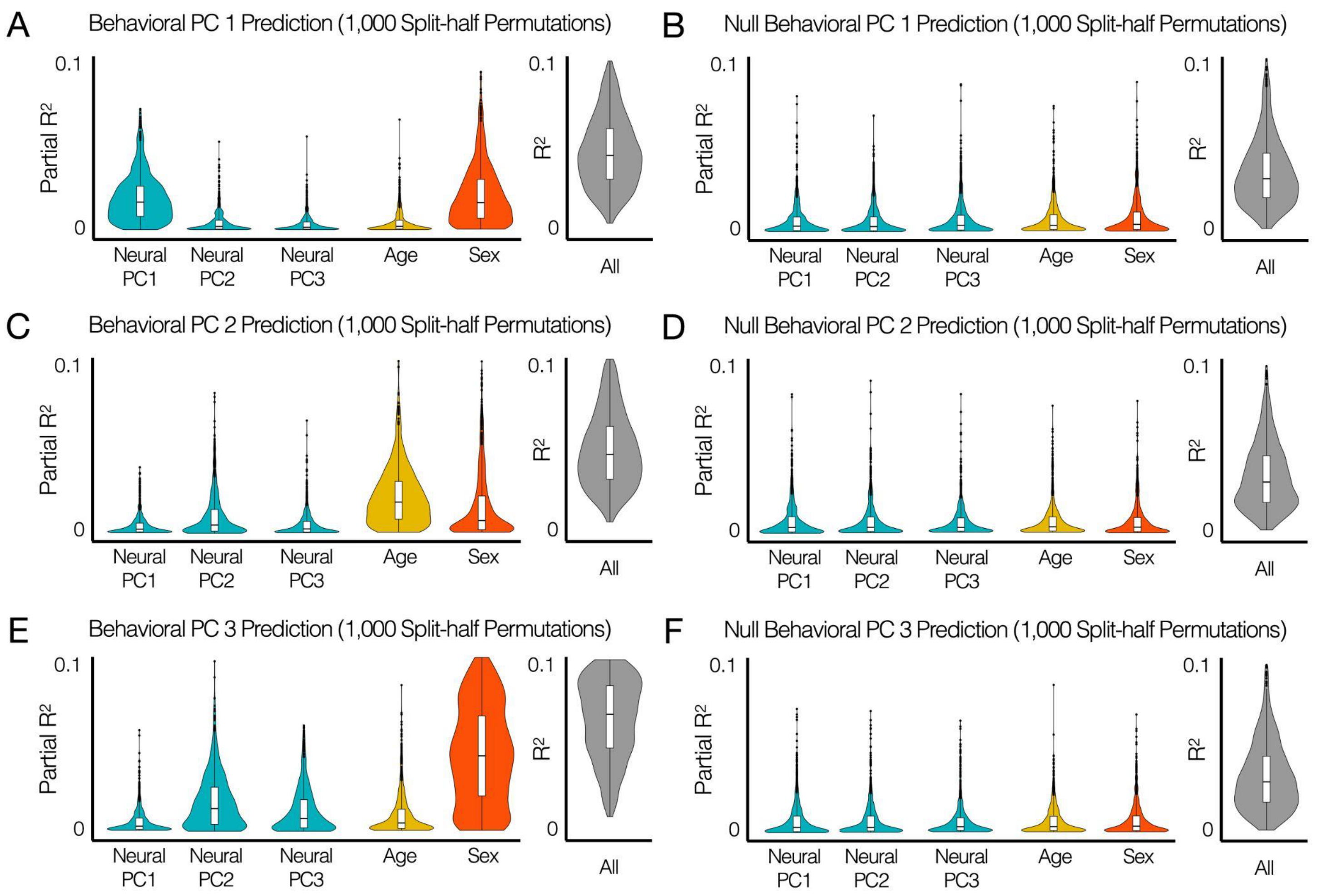

S17 Fig. Repeating the analysis of neuro-behavioral association excluding subjects with high motion did not change the results. Among 337 subjects, 28 subjects with excessive motion (FD>0.5 mm) were excluded. Across 1,000 permutations, a split of subjects (n=154) was randomly selected, and PCA was performed on neural measures from these subjects. Multiple linear regression models for predicting behavioral PCs from these subjects were estimated. Null data were generated by shuffling individual subjects in behavioral data.

Comment 6. Subcortical regions. It seems like subcortical regions were also included in the initial CAPs analysis, given that the Cole-Anticevic Brain Network Parcellation includes cortical surface and subcortical volume parcels. However, it is not clear whether the subcortical regions were included in the subsequent analysis. Could the authors comment on how subcortical data was handled and why it was excluded from the results?

Response: Thank you for your comments. We included both cortical and subcortical regions in all our analyses in this work. The patterns of co-activations in subcortical regions are illustrated in the basis set shown in Fig. 1, and all the following analyses were performed using this whole-brain CAPs. To clarify this, we revised the texts and Figure captions as follows.

(p.4) Results. “All 718 cortical and subcortical parcels were included in this and following analyses throughout this article.”

(Figure 2) Caption. “Note that all 718 cortical and subcortical parcels were included in this analysis. For simplicity, subcortical regions of CAPs are not visualized.”

(Figure 3) Caption. “Note that all 718 cortical and subcortical parcels were included in this analysis. For simplicity, subcortical regions of CAPs are not visualized.”

Response to Reviewer #4

Comment 1. The authors apply the well-studied CAP method to derive 4/5 canonical patterns and show that they are mostly reproducible in individual participants and that they are correlated with individual differences in behavior. Although the premise is interesting, I was not enthusiastic about the method, or whether the findings constitute a conceptual advance. As I outline below, I find it hard to believe that 15-minute chunks of neural recordings only contain information about 4/5 underlying processes. The patterns themselves do not appear to be highly differentiated from each other, or from the dominant PC1 in resting state FC.

Response: Thank you for the comments. In our study, we used 4 runs of 15-minute resting state fMRI data, therefore the total duration of data is about an hour. We revised the **Materials and methods** section by adding a significant amount of details about the data and analytic procedures, please refer to our response to Reviewers 2 and 3 for this matter. While it is true that there is substantial variability in individual co-activation patterns (**S7 Fig**), the estimated number of CAPs (the number of clusters) was low, e.g. 4 or 5, in our analysis. This is actually in agreement with other literature using similar approaches, as described in the revised **Materials and methods**. To determine the optimal number of clusters, we used an approach that considers a trade-off between the number of clusters and within-cluster similarity (e.g. silhouette criteria).

(p. 15) Methods. "Number of clusters. While the estimation of the number of clusters k is critical in CAP analysis, the field is lacking a consensus on the optimal criterion to determine it [70]. Earlier works used predefined arbitrary numbers ranging from 6 to 30 and reported that CAPs estimated using a small k are consistently found in results using a larger k [7,37,71]). It has been suggested that a large k may reduce cluster stability, for example, when a small number of time-frames are allocated to a cluster due to a short rs-fMRI acquisition duration [72]. Yang et al. calculated the silhouette scores for the clustering results varying k from 2 to 21 in both group and individual level analyses and chose k=4 as a trade-off to ensure the reliable estimation of spatiotemporal dynamics [55].

To find an optimal number, we used an approach that considers a trade-off between the number of clusters and within-cluster similarity (e.g. silhouette criteria [73]). We observed that the silhouette score was monotonically decreasing with the increase of k (**S2 Fig**), in agreement with Yang et al. [37]. In our study, to determine an optimal number of clusters, we varied the number of clusters (k) from 2 to 15. For each k, the K-means clustering was initialized using the k-means++ algorithm, by selecting randomly-generated centroids using sampling based on an empirical probability distribution of the points' contribution to the overall inertia. Inertia was defined as the sum of squared distances of samples to their closest cluster center. The maximum iteration for a single run was set to 1,000 to avoid that the algorithm stops before fully converging. Silhouette coefficient is estimated for the K-means solution using each k. Finally, an optimal number $\hat{k}$ is determined by applying the elbow method for the estimated Silhouette scores. The elbow point was defined when we observe a significant change in the rate of decrease of the Silhouette score as k increases, using the KneeLocator class in Python's kneed package. "

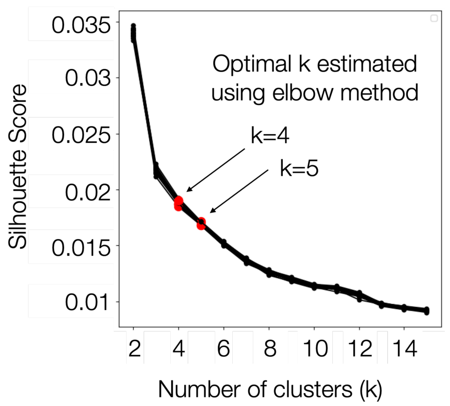

S2 Fig. Quality of the K means clustering solution in CAP analysis. Silhouette scores were estimated across different numbers of clusters (k) from the K-means clustering solution from a split data. Results from 10 permutations (two split-halves in each permutation) are shown. Optimal k values were estimated using the elbow method for the Silhouette scores and are highlighted in red.

(p. 4) Results. "The time-frames are clustered based on spatial similarity using K-means clustering, where the number of clusters (k) is estimated for each split using the elbow method varying k from 2 to 15 (see the estimated Silhouette scores from the K-means clustering solutions in (**S2 Fig**). "

In addition, we investigated the association of spatial variability of individual co-activation patterns (single time-frames) to the group level basis CAPs., as shown below.

(p.6) **Results. "Spatial alignment of individual time-frames to basis CAPs.** To find an optimal number of clusters or CAPs that are commonly found across individuals, we used an approach that considers a trade-off between the number of clusters and within-cluster similarity by combining the silhouette criteria and elbow method (**S2 Fig**). To evaluate the extent of the contribution of individual co-activation patterns to the observed CAP variability, we analyzed all fMRI time-frames obtained from 337 subjects after scrubbing. For each split, we computed the spatial alignment of individual 3-dimensional fMRI time-frames to the five basis CAPs (cluster centroids estimated by K-means clustering) using Pearson's correlation, identifying a basis CAP yielding the highest correlation with each time-frame. As a result, the mean and standard deviation of the maximum correlation were 0.22 ± 0.11 (**S8 Fig**), indicating the substantial variability in resting state human brain dynamics. Notably, the group-level spatial topography of CAPs, estimated by averaging the time-frames within each cluster, remained consistent across permutations (**Fig. 2**), enabling us to investigate individual differences in their temporal dynamics."

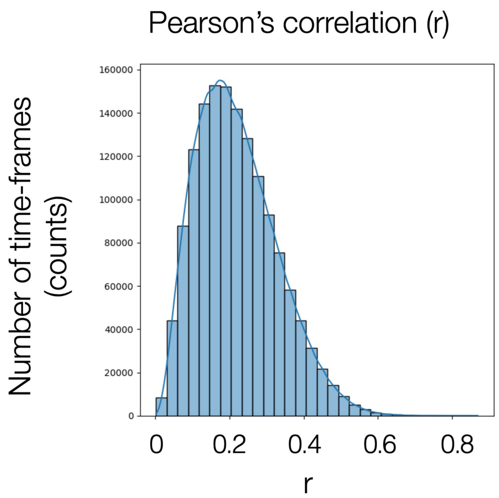

S8 Fig. The distribution of correlations between individual fMRI time-frames and the estimated basis CAPs (cluster centroid), to which individual time-frames were assigned by K-means clustering.

Our observation in **S8 Fig** suggests that individual co-activation time-frames involve a substantial amount of variability. Traditionally, the estimation and analysis of CAPs have been implemented at the group level, and the field is lacking a robust framework to quantify reproducible spatial patterns of individual co-activation and derive an interpretable and reproducible neuro-behavioral relationship. A recent work by Yang et al., (2023) suggests a method to detect individual CAPs at the subject-level by maximizing individual identifiability by measuring the gap between within-subject and between-subject similarity. We discussed this in the revised manuscript as follows.

(p. 16) Discussion. "A recent work suggests a method to detect individual CAPs at the subject-level by maximizing individual identifiability [55]. Using the densely sampled Midnight Scan Club dataset [56], they identified four CAPs at both the group-level and single-subject level, with two CAP pairs exhibiting opposing spatial patterns, similar to the findings in this work (**Fig. 1**). However, their study did not address how individualized CAPs and their temporal profiles can be assessed in relation to behavior, focusing on an identical number of clusters at both levels. In contrast, we focused on identifying reproducible spatial patterns of neural co-activation (CAPs) at the group level and quantifying a reproducible feature set of temporal profiles of the CAPs at the single subject level, therefore providing a unified framework to evaluate individual differences using a joint analysis of state and trait variations of neural co-activation. Similarly, several brain states were estimated at the group level using another dynamic functional connectivity approach in [24], whereas they could identify the time-points when the state was active, allowing the estimation of FO for each state and for each subject.

Collectively, these results highlight that state-trait CAP dynamics are reproducible at the single subject level across permutations and splits (**Fig. 3, S9 Fig**). For context, the statistics reported here (**Fig. 3C**) demonstrate higher reproducibility than the meta-analytic estimate for group-level reproducibility of area-to-area functional connectivity matrices [51]. Still, the observed ICC values are fairly low, reflecting a notable variance between days. This indicates that while trait-like features are most dominant factors loaded on the general motif of individual variance, the observed state variance (between days) also contributes to this general motif. In this context, the fairly low range of ICC reflects the notable amount of within-subject variance at the time-scale of days, and supports our framework of state-trait components that together identify neural phenotypes. Similarly,

Yang et al. found that CAPs identified at individual level were unstable over time across the ten scans (~ 30 minutes/scan) except a few subjects, and subject-specific CAPs became more reliable and individual specific when integrating data with longer duration [55]. Therefore, we argue that such temporal fluctuations within subjects can bring critical insights into individual-specific brain organizations. Reducing the number of neural features into a reproducible set of CAPs may enable a more robust and reproducible mapping between neural features and behavior. In other words, we hypothesize that further optimization of reproducible data-reduced neural features presents a critical step toward mapping rs-fMRI signals to healthy and clinically-relevant behavioral variation and obtaining robust neuro-behavioral models."

Comment 2. Why make the assumption that only one CAP is present in any single volume? Surely multiple sensory, cognitive and motor processes can take place simultaneously.

Comment 3. On a related note, the authors find that there are an average of 4-5 CAPs per participant. This, like other discrete-state methods used in neuroscience, strikes me as counter-intuitive, in the sense that are probably many many more simultaneous processes taking place during an average 15-minute scan.

Response: We appreciate your comments about the potential multiple brain states that may overlap in each single time-frame. Indeed, there are other dynamic functional connectivity approaches assuming multiple brain states in each time point, in contrast to our approach and other discrete-state methods. It is unknown, either theoretically or experimentally, whether the changes in brain dynamics involve or best represented by discrete or overlapping states. It was beyond the scope of this study to argue that the brain states are discrete in time, however, we were rather interested in quantifying neural features that vary within subjects (state features) and between subjects (trait features) and analyzing them together in a single framework to understand how individual differences in neural state-state features can be reproducibly mapped to behavior. Although, we understand the concern raised by the reviewer, because the fundamental assumption about brain states can impact the interpretation about the results and the neurobiological underpinnings of these findings. We addressed this by adding more literature and discussing them with our current work, as follows.

Updated References.
[24] Vidaurre, Smith & Woolrich,Brain network dynamics are hierarchically organized in time, PNAS, 2017
[25] Taghia et al., Uncovering hidden brain state dynamics that regulate performance and decision-making during cognition, Nat Communications, 2018
[26] Shine et al., Human Cognition involves the dynamic integration of neural activity and neuromodulatory systems, Nat Neuroscience 2019
[61] Larsen et al., A critical period plasticity framework for the sensorimotor-association axis of cortical development, Trends in Neuroscience, 2023
[62] Luo et al., Functional connectivity development along the sensorimotor-association axis enhances the cortical hierarchy, Nature Communications, 2024

(p. 2) Introduction. " The analyses of neural state dynamics or time-varying rs-fMRI connectivity can be used to understand individual differences [24]. Evaluating moment-to-moment changes in neural activity can provide information about latent brain states associated with task-switching and decision-making in working memory [25]. Using dimension reduction of task fMRI data across multiple cognitive tasks, Shine et al. suggested that execution of diverse cognitive tasks and individual differences in fluid intelligence can be described using a

dynamic flow along a low-dimensional manifold of global brain activity [26]. There is a knowledge gap regarding how combined state and trait variation of spontaneous brain dynamics map onto individual variation in complex behavioral phenotypes."

(p.7) Results. "In this study, we are interested in testing the hypothesis that there is a reproducible general motif of individual differences in neural co-activation dynamics, where individuals differently occupy (or project onto). While previous work in [26] focused on a low-dimensional manifold of spatiotemporal neural activity by applying principal component analysis of rs-fMRI signal volumes, we aim to identify a low-dimensional feature space that characterizes state and trait properties of the temporal organizations of brain states. To do this, we first demonstrate that state-trait CAP dynamics are reproducible at the single subject level across permutations, whereas within-subject between-day reliability was lower than between-permutation reliability on a same day (**Fig. 3B**, **C**, **S10 Fig**)"

(p. 16) Discussion. "Similarly, several brain states were estimated at the group level using another dynamic functional connectivity approach in [24], whereas they could identify the time-points when the state was active, allowing the estimation of FO for each state and for each subject. **"**

(p.18) Discussion. "Our results converge with several findings using other dynamic functional connectivity approaches. Vidaurre et al. used Hidden Markov Model to identify 12 brain states using the HCP-YA rs-fMRI datasets and derived two metastates, each being a set of brain states that are more likely to transit between each other [24]. They found that the FO of brain states and their associated metastates are subject-specific and behaviorally relevant, highlighting several well-being, intelligence and personality traits [24]. It would be interesting to evaluate, using the same subjects studied in this work, if brain activity during cognitive tasks exhibit a similar set of CAP features and low-dimensional state-trait variances to those estimated using rs-fMRI and how they are related to such high-order metastates [24] and the low-dimensional global brain activity found across multiple cognitive tasks [26].**"**

Comment 3. Many of the CAPs are highly anatomically similar, and also generally similar to the frequently-studied principal gradient of FC. There is quite a bit of attention on resting state networks but clearly the FC PC1 is the dominant signature in all these patterns. More generally, it is unclear what these patterns represent biologically. Are they related to the underlying micro- or macro-scale anatomy?

Response: Thank you for the comment. To enrich the neuroscientific relevance and interpretation of our findings, we first extended our analysis to associate the first three neural PCs and the first three behavioral PCs. We ensured the reproducibility of our neuro-behavioral mapping using permutation and cross-validation (see **Figure 6, S16 Fig** and **S17 Fig**). Then, we discussed these results in relation to underlying biology.

Additional References:

[61] Larsen et al., A critical period plasticity framework for the sensorimotor-association axis of cortical development, Trends in Neuroscience, 2023

[62] Luo et al., Functional connectivity development along the sensorimotor-association axis enhances the cortical hierarchy, Nature Communications, 2024

(p. 19) Discussion. "Our results in the reproducible neuro-behavioral associations highlighted the neural relevance of behavioral PCs 1, 2 and 3. Individuals with higher cognition (behavioral PC 1) spent longer time at CAP I than at CAP II. The behavioral PC 2 showed an association with neural PCs 1 and 2, as well as age and sex. In relation to resting state functional connectivity, While CAPs I and II show differences in the regions of language, primary visual, and cingulo-opercular networks, the overall patterns of these CAP states involved opposing patterns of brain activity in the default mode and frontoparietal networks versus the regions belonging to the secondary visual, somatomotor and dorsal attention networks [61]. This suggests a relation to the cortical hierarchy that spans from unimodal sensorimotor cortices to transmodal association cortices and a potential role in functional connectivity development during childhood through adolescence [62]."

Comment 4. The motivation presented in the Introduction suggests that CAPs could better capture individual differences in behavior because they are sensitive to both state and trait. Yet there is no clear comparison between the two.

Response: We agree that the present work does not directly compare the performance of neuro-behavioral mapping when using only state features, only trait features and both state and trait features. Indeed, the investigation on whether the state and trait neural features reflect unique or additive information about individual differences is an important future direction. We addressed this in our discussion as follows.

(p. 17) Discussion. "While this study suggests the importance of taking state variations into account when studying neural basis of individual differences, we did not directly compare the performance of neuro-behavioral mapping when using only state features, only trait features and both state and trait features. Further studies with comprehensive experiments on state manipulations such as pharmacological neuroimaging or transcranial magnetic stimulation (TMS) can help to understand how state and trait neural features change in response to such manipulations and whether the state and trait neural features reflect unique or additive information about individual differences."
