# Supplementary material for: Human brain state dynamics are highly reproducible and associated with neural and behavioral features
Source: PLoS Biol. 2024 Sep 24;22(9):e3002808. doi: 10.1371/journal.pbio.3002808 (PMC11421804; doi:10.1371/journal.pbio.3002808)
Supplement: Attachment — Submitted filename: Response_to_Reviews_cap_PLOS_Biology_R3.pdf [file pbio.3002808.s024.pdf]

Response to Reviewers

Ms. No.: PLOS Biology
Title: **Human brain state dynamics are highly reproducible and associated with neural and behavioral features**
Corresponding Authors: Dr. Kangjoo Lee and Dr. Alan Anticevic
Authors: Dr. Kangjoo Lee, Dr. Jie Lisa Ji, Dr. Clara Fonteneau, Dr. Lucie Berkovitch, Dr. Masih Rahmati, Lining Pan, Dr. Grega Repovš, Dr. John H. Krystal, Dr. John D. Murray, Dr. Alan Anticevic

Dear editors and reviewers:

We would like to thank the editors for the opportunity to revise and resubmit our paper and the reviewers for their additional comments. The additional questions raised by the reviewers 3 and 4 are addressed below. Comments from the reviewer and any unchanged texts in the revised manuscript are written in "**black**". Our responses to comments are written in "**blue**" and any changes in the revised manuscript are highlighted in "**red**". We also addressed policy-related requests to meet the criteria of PLOS Biology. We hope the reviewers and editors find the revised manuscript satisfactory.

Response to Reviewer #3 (Golia Shafiei & Ted Satterthwaite)

Comment 1. All of our comments have been largely addressed. One final point that could merit clarification (but should not delay in publication) is in regards to scrubbing. First, Does not appear to be described in the (appropriately expanded) new method section. Second, it is somewhat unclear how a scrubbing interval interacts with measures such as dwell time. If a consistent state is interrupted by a high motion volume and scrubbing is applied, is that considered a state transition, or does the dwell time continue? While the authors' analyses suggest that it is unlikely that these scrubbing events impact the results, such methodological details would be nice for readers to be able to refer to.

Response: Thank you for the reviewers and we now better understand your comments. We added a new section in Methods and described the methodological details for potential bias in the estimation of dwell time.

(p. 23) Methods. "Quantification of neural measures and the impact of motion. Fractional occupancy ((FO(***s***,***i***)), is defined as the total number of time-frames (# TR) that a subject ***s*** spends in CAP state ***i*** per day, normalized by the total number of time-frames spent in any CAP state by subject ***s*** per day. Dwell time ((DT(***s***,***i***,***c***))) is defined as the number of time-frames (# TR) of a time-consecutive segment ***c*** occupying the same CAP state ***i*** within a subject ***s*** per day. Within-subject mean and variance of DT, Mean DT(***s***,***i***)) and Var DT(***s***,***i***), were computed as the mean and standard deviation of estimated values of DT from all time-consecutive segments occupying a CAP ***i*** within a subject ***s*** per day. These CAP measures were estimated for each split data per permutation and for each day separately. Note that time-frames with excessive motion (FD >0.5 mm) were scrubbed before computing these CAP measures [48,69]. Motion scrubbing is helpful in minimizing the deleterious impacts of motion on the analysis of CAPs and CAP-derived measures, but it can potentially affect the quantification of these CAP measures [75]. While CAP states during motion-related time-frames remain unknown because scrubbing is performed before the CAP identification, the time-frames before and after the motion-related time-frames would be combined into a single consecutive segment if they occupy the same CAP state. For example, if a consistent CAP state (e.g. [I,I,I], true DT=3) is interrupted by a high motion volume M (e.g. [I,M,I]), the estimated DT would be shorter than the true value ([I,I], estimated DT=2). Conversely, if the CAP state during motion differs from that before and after ([I,II,I], true DT=1 for each of three segments), DT would be estimated for the combined segment after scrubbing [I,I], resulting in DT=2. To verify that our findings were not impacted by this effect, we investigated the amount of motion (number of scrubbed time-frames) and the duration of motion (number of consecutive time-frames with excessive motion). We identified subjects whose fMRI time-series involved relatively high motion, resulting in the scrubbing of more than 5% of total time-frames, and repeated the whole analyses excluding these subjects."

Added a new reference:

[75] Bolton et al. "TbCAPs: A toolbox for co-activation pattern analysis", Neuroimage, 2010; 211: 116621

Response to Reviewer #4

Comment 1. The authors have addressed my concerns to the best of their knowledge, and in line with the standards in the field. I am therefore happy to defer to the Editor and other reviewers. However, I still believe that the biological interpretation of these CAPs is ambiguous and that the assumptions imposed (a small number of discrete states) are unrealistic.

Response: We very much appreciate the Reviewer's overall positive feedback and acknowledgment of our attempt to address all of their suggestions and comments. We also hear the Reviewer's continued impression that the biological interpretation of CAPs is perhaps not entirely clear. To address this, we have added the following section to the Discussion, in which we also discuss the choice of the number of discrete states.

(p. 19-20) Discussions. "**Biological interpretation of CAPs.** In general, CAPs derived from BOLD fMRI signals can be interpreted as patterns of how different brain regions synchronize and coordinate their activity over time. The three CAPs identified in this study represent brain-wide co-activation motifs that can be reproducibly observed in the general populations at the given temporal and spatial resolution. Although these three CAPs, with positive and negative signs, may not encompass all possible brain states, it is notable that our result converges on such a reproducible low number. While the CAP analysis is dependent on the number of samples (subjects) and time-points, in this study, this limitation was balanced by determining the number of CAPs by what was found to be reproducible. However, we acknowledge that the true number of CAPs in the human brain may exceed what is established in this analysis. Future research using larger datasets could reveal additional reproducible brain states, including rare but consistent CAPs. Potential factors contributing to individual variability during rs-fMRI scans, such as extreme anxiety, heightened emotion, ongoing cognition or physical pain during resting-state scans, might impact CAP estimation. Investigating this variability in detail could help identify potential motifs that were previously considered rare or non-reproducible in smaller samples, but may become evident in larger datasets, such as the full samples from HCP-YA S1200 dataset or UK Biobank [45]."

Added a new reference:

[45] Bycroft C et al. The UK Biobank resource with deep phenotyping and genomic data. Nature. 2018;562:(7726):203–209.

Response to Policy-related Requests

Request 1. We would like to suggest a different title to improve its accessibility: "Human brain state dynamics are highly reproducible and associated with neural and behavioral features"

Response: We revised the title of the manuscript as suggested.

Title: Human brain state dynamics are highly reproducible and associated with neural and behavioral features

Request 2. Please add the links to the funding agencies in the Financial Disclosure statement in the manuscript details.

Response: We added the URL of each funder's website as follows in the submission system.

"This work was supported by 5P50AA012870-22 SP-National Institute on Alcohol Abuse and Alcoholism (NIAAA)/NIH/DHHS (https://www.niaaa.nih.gov/) for J.H.K. and 5U01MH121766-03 SP-National Institute of Mental Health (NIMH)/NIH/DHHS (https://www.nimh.nih.gov/) for A.A. L.B. was supported by the Fondation Bettencourt Schueller (https://www.fondationbs.org/en) and the Philippe Foundation (https://www.philippefoundation.org/). G.R. was supported by the Slovenian Research Agency (ARRS) grants P3-0338, J7-8275, J5-4590 (http://www.aris-rs.si/en/). The funders had no role in study design, data collection and analysis, decision to publish, or preparation of the manuscript."

Request 3. All research involving human participants must have been conducted according to the principles expressed in the Declaration of Helsinki. Please state in the manuscript that this is the case for your study (if correct).

Response: We stated this in the revised manuscript as follows.

(p. 24) Ethics Statement. In the collection of HCP Young Adult S1200 data, each participant provided their review and signature on the informed consent document at the start of day 1, as directed by the institutional review board (IRB) at Washington University at St. Louis, USA (45). This study was conducted according to the principles expressed in the Declaration of Helsinki. K.L, J.L.J. and A.A. have obtained the acceptance of HCP Open Access Data Use Terms for access to all HCP data. K.L further obtained the approval for access to Restricted Data generated by HCP, WU-Minn-Ox HCP. All analyses conducted in this work were approved by the IRB at Yale University (IRB number: 1111009332), Connecticut, USA. This work is 100% based on human effort, and no artificial intelligence (AI)-assisted technologies were used in the production of this article.

In addition, the updated Conflict of interests reflects the first author KL's new consulting role.

(p. 24) Declarations. K.L. consults for Manifest Technologies. A.A. and J.D.M. hold equity with Neumora Therapeutics (formerly BlackThorn Therapeutics), Manifest Technologies, and are co-inventors on the following patents: Anticevic A, Murray JD, Ji JL: Systems and Methods for Neuro-Behavioral Relationships in Dimensional Geometric Embedding(N-BRIDGE), PCT International Application No.PCT/US2119/022110, filed March 13, 2019 and Murray JD, Anticevic A, Martin WJ: Methods and tools for detecting, diagnosing,

predicting, prognosticating, or treating a neurobehavioral phenotype in a subject, U.S. Application No.16/149,903, filed on October 2, 664 2018, U.S. Application for PCT International Application No.18/054, 009 filed on October 2, 2018. J.L.J. is an employee of Manifest Technologies, has previously worked for Neumora, and is a co-inventor on the following patent: Anticevic A, Murray JD, Ji JL: Systems and Methods for Neuro-Behavioral Relationships in Dimensional Geometric Embedding (N-BRIDGE), PCT International Application No.PCT/US2119/022110, filed March 13, 2019. C.F. consults for Manifest Technologies and formerly consulted for RBNC (formerly BlackThorn Therapeutics). G.R. consults for and holds equity in Neumora and Manifest Technologies. L.P. is an employee of Manifest Technologies. J.H.K. holds equity in Biohaven Pharmaceuticals, Biohaven Pharmaceuticals Medical Sciences, Clearmind Medicine, EpiVario, Neumora Therapeutics, Tempero Bio, Terran Biosciences, Tetricus, and Spring Care. J.H.K. consults for AE Research Foundation, Aptinyx, Biohaven Pharmaceuticals, Biogen, Bionomics, Limited (Australia), BioXcel Therapeutics, Boehringer Ingelheim International, Cerevel Therapeutics, Clearmind Medicine, Cybin IRL, Delix Therapeutics, Eisai, Enveric Biosciences, Epiodyne, EpiVario, Evidera, Freedom Biosciences, Janssen Research \& Development, Jazz Pharmaceuticals, Leal Therapeutics, Neumora Therapeutics, Neurocrine Biosciences, Novartis Pharmaceuticals Corporation, Otsuka America Pharmaceutical, Perception Neuroscience, Praxis Precision Medicines, PsychoGenics, Spring Care, Sunovion Pharmaceuticals, Takeda Industries, Tempero Bio, Terran Biosciences, and Tetricus. All other co-authors declare no competing interests.

Request 4. DATA POLICY:

You may be aware of the PLOS Data Policy, which requires that all data be made available without restriction: https://nam12.safelinks.protection.outlook.com/?url=http%3A%2F%2Fjournals.plos.org%2Fplosbiology%2Fs%2Fdata-availability&data=05%7C02%7Ckangjoo.lee%40yale.edu%7C39048de7bd124579783f08dca81c460f%7Cdd8cbebb21394df8b4114e3e87abeb5c%7C0%7C0%7C638570086591082037%7CUnknown%7CTWFpbGZsb3d8eyJWIjoiMC4wLjAwMDAiLCJQIjoiV2luMzIiLCJBTiI6Ik1haWwiLCJXVCI6Mn0%3D%7C0%7C%7C%7C&sdata=azrsGqUKDuL5YWDPbDw04bTSRf4Yo5STVSKUUowLqFA%3D&reserved=0. For more information, please also see this editorial: https://nam12.safelinks.protection.outlook.com/?url=http%3A%2F%2Fdx.doi.org%2F10.1371%2Fjournal.pbio.1001797&data=05%7C02%7Ckangjoo.lee%40yale.edu%7C39048de7bd124579783f08dca81c460f%7Cdd8cbebb21394df8b4114e3e87abeb5c%7C0%7C0%7C638570086591092171%7CUnknown%7CTWFpbGZsb3d8eyJWIjoiMC4wLjAwMDAiLCJQIjoiV2luMzIiLCJBTiI6Ik1haWwiLCJXVCI6Mn0%3D%7C0%7C%7C%7C&sdata=EhlQoRTkSuK20%2B%2BJ%2F6v1DWZZyxSq%2BIHzGCJlN%2BCAmSA%3D&reserved=0

Note that we do not require all raw data. Rather, we ask that all individual quantitative observations that underlie the data summarized in the figures and results of your paper be made available in one of the following forms:

1) Supplementary files (e.g., excel). Please ensure that all data files are uploaded as 'Supporting Information' and are invariably referred to (in the manuscript, figure legends, and the Description field when uploading your files) using the following format verbatim: S1 Data, S2 Data, etc. Multiple panels of a single or even several figures can be included as multiple sheets in one excel file that is saved using exactly the following convention: S1_Data.xlsx (using an underscore).

2) Deposition in a publicly available repository. Please also provide the accession code or a reviewer link so that we may view your data before publication.

Regardless of the method selected, please ensure that you provide the individual numerical values that underlie the summary data displayed in the following figure panels as they are essential for readers to assess your analysis and to reproduce it: 3C, 5C, 6DEFGH, 7A and similar panels in the supplementary figures.

NOTE: the numerical data provided should include all replicates AND the way in which the plotted mean and errors were derived (it should not present only the mean/average values).

Please also ensure that figure legends in your manuscript include information on where the underlying data can be found, and ensure your supplemental data file/s has a legend.

Please ensure that your Data Statement in the submission system accurately describes where your data can be found.

CODE POLICY

Per journal policy, if you have generated any custom code during the course of this investigation, please make it available without restrictions. Please ensure that the code is sufficiently well documented and reusable, and that your Data Statement in the Editorial Manager submission system accurately describes where your code can be found.

Please note that we cannot accept sole deposition of code in GitHub, as this could be changed after publication. However, you can archive this version of your publicly available GitHub code to Zenodo. Once you do this, it will generate a DOI number, which you will need to provide in the Data Accessibility Statement (you are welcome to also provide the GitHub access information). See the process for doing this here: https://nam12.safelinks.protection.outlook.com/?url=https%3A%2F%2Fdocs.github.com%2Fen%2Frepositories%2Farchiving-a-github-repository%2Freferencing-and-citing-content&data=05%7C02%7Ckangjoo.lee%40yale.edu%7C39048de7bd124579783f08dca81c460f%7Cdd8cbebb21394df8b4114e3e87abeb5c%7C0%7C0%7C638570086591097598%7CUnknown%7CTWFpbGZsb3d8eyJWIjoiMC4wLjAwMDAiLCJQIjoiV2luMzIiLCJBTiI6Ik1haWwiLCJXVCI6Mn0%3D%7C0%7C%7C%7C&sdata=3cWziC67PXwPtk97LbuTPAHQgvMIGvHFjfTqLDgZzf4%3D&reserved=0

* Please note that per journal policy, we do not allow the mention of "data not shown", "personal communication", "manuscript in preparation" or other references to data that is not publicly available or contained within this manuscript. Please either remove mention of these data or provide figures presenting the results and the data underlying the figure(s).

Response: We submit Supplementary Information files underlying the Figures 3, 5, 6, 7, S15 Fig , S16 Fig and S17 Fig, as follows.

S1 Data. Data used to generate Figure 3(C). Three datasets for fractional occupancy, within-subject mean and standard deviation of dwell time are provided. Column A: CAP identification (0: CAP I+, 1:CAP I-, 2: CAP II+, 3: CAP II-, 4:CAP III). Column B: Split 1 or 2. Column C: Permutation index (1-1000), Column D: Intraclass Correlation Coefficient (ICC).

S2 Data. Data used to generate Figures 5 and 6. Sheet 1 (Behavioral PC Loadings) involves the loadings of 262 behavioral variables (columns) on the behavioral PCs 1-15 (rows). Sheet 2 (Individual PC scores) involves the scores of individual subjects (rows) for the behavioral PCs 1-15 (columns).

S3 Data. Data used to generate Figure 6, S15 Fig and S16 Fig. (Sheets 1-3) Results in Figure 6 and S16 Fig. (Sheet 4-6) Null data results in S15 Fig and and S16 Fig. Columns A-G involve results from the first analysis using split-half datasets across 1,000 permutations. Column A (permutation index). Columns B-F: Partial R^2 estimated for neural PC 1, neural PC 2, neural PC 3, age and sex. Column G: Overall R^2. Columns H-J involve results from the second analysis using cross-validation, where the multiple linear regression model from Split 1 data was used for predicting individual behavioral PC scores in the corresponding split 2 data across permutations. Column H: Correlation coefficient. Column I: p-value estimated for the correlation coefficient. Column J: Overall R^2.

S4 Data. Data used to generate Figure 7. Column A (subgroup_30nf): Subgroup identified for each individual using 30 neural features. Column B (subject ID). Column C (k5-k4_counts_mean12_z): Probability of CAP III occurrence (z). Column D-F. Individual scores on neural PCs 1-3. Column G. Individual scores on behavioral PC 1.

S5 Data. Data used to generate S17 Fig. (Sheets 1-3) Results excluding subjects with high motion. (Sheet 4-6) Null data results excluding subjects with high motion.

Fig 3. "The data used to generate the results can be found in **S1 Data**."
Fig 5. "The data used to generate the results can be found in **S2 Data**."
Fig 6. "The data used to generate the results can be found in **S2 Data** and **S3 Data**."
Fig 7. "The data used to generate the results can be found in **S4 Data**."
S16 Fig. "The data used to generate the results can be found in **S3 Data**."
S17 Fig. "The data used to generate the results can be found in **S5 Data**."

Finally, we included the following text in the section "**Data Availability**" of the main manuscript file and the **Data Accessibility Statement** on the submission system**.** In addition, the codes used in this study have been published on Zenodo as per the guideline.

Data Availability. All relevant raw data files are available from the Human Connectome Project (HCP) S1200 data database (https://www.humanconnectome.org/study/hcp-young-adult/document/1200-subjects-data-release). The datasets underlying the results in this work are provided in Supporting Information files. The codes used in this study are available on Zenodo: Lee, K. (2024). pyCAP codes: Human brain state dynamics are highly reproducible and associated with neural and behavioral features. Zenodo. https://doi.org/10.5281/zenodo.13251562.
